# Defining Individual-Level Genetic Diversity and Similarity Profiles

**DOI:** 10.1038/s41598-020-62362-8

**Published:** 2020-04-02

**Authors:** Zhanshan (Sam) Ma, Lianwei Li, Ya-Ping Zhang

**Affiliations:** 1 Computational Biology and Medical Ecology Lab, State Key Laboratory of Genetic Resources and Evolution, Kunming Institute of Zoology, Chinese Academy of Sciences, Kunming, 650223 China; 2 Molecular Evolution and Genome Diversity Lab, State Key Laboratory of Genetic Resources and Evolution, Kunming Institute of Zoology, Chinese Academy of Sciences, Kunming, 650223 China; 3 Center for Excellence in Animal Evolution and Genetics, Chinese Academy of Sciences, Kunming, 650223 China

**Keywords:** Computational biology and bioinformatics, Molecular biology

## Abstract

Classic concepts of genetic (gene) diversity (heterozygosity) such as Nei & Li’s nucleotide diversity were defined within a population context. Although variations are often measured in population context, the basic carriers of variation are individuals. Hence, measuring variations such as SNP of an individual against a reference genome, which has been ignored previously, is certainly in its own right. Indeed, similar practice has been a tradition in community ecology, where the basic unit of diversity measure is individual community sample. We propose to use Renyi’s-entropy-based Hill numbers to define *individual-level* genetic diversity and similarity and demonstrate the definitions with the SNP (single nucleotide polymorphism) datasets from the 1000-Genomes Project. Hill numbers, derived from Renyi’s entropy (of which Shannon’s entropy is a special case), have found widely applications including measuring the quantum information entanglement and ecological diversity. The demonstrated individual-level SNP diversity not only complements the existing population-level genetic diversity concepts, but also offers building blocks for comparative genetic analysis at higher levels. The concept of *individual* covers, but is not limited to, individual chromosome, region of chromosome, gene cluster(s), or whole genome. Similarly, the SNP can be replaced by other structural variants or mutation types such as indels.

## Introduction

SNPs (single nucleotide polymorphism) are single-nucleotide substitutions of one base for another and arguably the commonest genetic variation. There are two general categories of approaches to investigating SNPs: one is the genomic approach and another is the functional approach. With genomic approach, scientists have catalogued the SNP database in the 3-billion-base pair human genome (*e.g*., https://www.ncbi.nlm.nih.gov/snp/, http://www.hgvs.org/central-mutation-snp-databases). The functional approaches have been adopted by scientists and clinicians who are interested in the implications of SNPs to a particular disease or drug response. With either approaches, statistically characterizing the *abundance* and *distribution* of SNPs is both important but challenging. Several existing characterizations of SNPs have been developed including: computing heritability (*e.g*. Yang *et al*.)^1^, computing gene and pathway scores to improve statistical power and gain biological insight (*e.g*. Lamparter *et al*.)^2^, genetic variation analysis (*e.g*., The Genomes Project Consortium, 2015)^3^, and distribution fitting (*e.g*. Tang *et al*.)^4^.

Amos^5^ found that the distributions of even small SNP clusters are non-randomly distributed in the human genome^5^. In other words, SNPs are not distributed at random across the chromosome or whole genome, but are aggregated or clustered. A variety of processes from ascertainment biases (*i.e*., the preferential development of SNPs around interesting genes) to the action of mutation *hot spots* and natural selection may be responsible for the highly non-random distribution of SNPs. For example, natural selection may modulate local variability along a chromosome to generate non-randomness. The distribution of SNPs along a chromosome is frequently harnessed to infer the action of natural selection. The non-random distribution of SNPs has far reaching ramifications for how to statistically characterize SNPs properly, in particularly, the choice of summary statistics. For example, the non-random and highly skewed distribution (Amos)^5^ of the SNP makes many of the commonly used *aggregation* functions such as arithmetic mean (average) and even median poor metrics for characterizing SNPs (*e.g*., Beliakov *et al*., James)^6,7^. Instead, the entropy-based *aggregation functions* such as Shannon’s entropy and Renyi’s general entropy should be more appropriate for summarizing the information transpired by SNPs. In fact, Shannon entropy, which was borrowed from Shannon^8^ information theory, has been the most widely used metric for measuring species diversity (also known as ecological diversity, community diversity or biodiversity), although recent studies (Chao *et al*. Jost, Ellison)^9–12^ have reached a consensus that the Hill numbers, which are derived from Renyi’s general entropy, offer the most appropriate alpha-diversity metrics, and are advantageous for multiplicatively partitioning beta-diversity. In following sections, we will define the SNP diversity with Hill numbers and obtain a series of indexes for summarizing the distribution of SNPs.

Of course, measuring diversity with entropy is not new at all, and the concepts of genetic (gene) diversity (heterozygosity) have been proposed and widely applied since pioneering works in the 1970s (Nei 1973, Nei & Li 1979)^13,14^. We observed that all existing genetic (gene) diversity have been defined within the context of population. Although variations are often measured in population context, the basic carriers of variation are individuals. Hence, measuring variations such as SNP of individual against a reference genome, which has been ignored currently, is certainly in its own right. Indeed, similar practice has been the tradition in ecology, where the basic framework of diversity measure is individual community sample. We fill this gap in existing literature of genetic (gene) diversity by learning from ecology to define individual-level genetic diversity and similarity profiles using the SNP as an example.

In ecology, Hill numbers (Hill)^15^ capture the essential properties of species abundance distribution (SAD) in a community and hence provide effective metrics for measuring species diversity because SAD contains full diversity information about a community. Hill numbers were derived from Renyi^16^ general entropy, of which Shannon entropy is a special case, and which has found wide applications in various fields of science and technology, from measuring quantum information entanglement to the wealth distribution in economics, and more recently from measuring ecological diversity (*e.g*., Golan 2008, Hastings *et al*., Chao *et al*., Kaplinsky & Arnaout)^9,10,17–19^ to measuring metagenome diversity (Ma & Li)^20^. As reiterated in Sherwin *et al*.^21^, information theory has been playing a broadening role in molecular ecology and evolution. Similar to their critical roles in measuring ecological diversity, Hill numbers can capture essential properties of the SNP distribution on a genetic entity such as a chromosome or a genome and offer effective metrics for measuring SNP diversity.

The primary objective of this article is to define the genetic diversity at the individual level using SNP as prototype, but the definitions can be equally applicable to other types of mutations commonly measured in the context of genetic diversity. Those additional types include but not necessarily limited to: deletion, duplication, inversion, insertion, translocation *etc*, but SNP is by far the commonest type. We will define the SNP diversity with Hill numbers at the individual level, including the alpha diversity, beta diversity, and gamma diversity of SNPs. Of course, to define SNP of an individual, a reference genome is required. Therefore, to define SNP diversity, two individuals including a reference genome and a target genome are required. In contrast, existing concepts (indexes) of genetic (gene) diversity were all defined in a population of more than two individuals. The SNP alpha-diversity we will define, in effect, measures the unevenness or heterogeneity of SNPs in a genetic entity such as a chromosome or a genome at the individual level. This not only complements the current population-level genetic (gene) diversity, but also provides building blocks for further comparative SNP analyses. For example, our SNP beta-diversity is defined to measure the difference between two or more individuals, and SNP gamma diversity is defined to measure the total diversity within the individuals of a population. Therefore, our concept and supporting metrics of SNP diversity provide a cross-scale tool for analyzing SNP variations at both individual and population levels.

We also define four SNP similarity metrics based on the Hill numbers. The SNP similarity metrics can be utilized to directly compare the SNP distribution patterns of the so-termed *N*-population, *i.e*., a population or cohort consisting of *N* individuals. Together, SNP diversity and similarity measures in Hill numbers offer effective tools to reveal genetic and evolutionary insights SNPs may reveal. We demonstrate the implementations (computation) of our definitions for the SNP diversity and similarity measures with the SNP datasets obtained from 1000-Genomes Project, consisting of 2504 individuals belonging to 5 populations (The 1000 Genomes Project Consortium 2015; Sudmant *et al*.)^3,22^.

As a side note, our title used the term “profile” (of diversity/similarity), which is to do with the definitions of Hill numbers. Hill numbers (also termed *diversity profile*) are a series of diversity measures that are weighted differently by the occurrences of low frequency SNPs, which form the long tail of the highly skewed SNP distribution and is often responsible for the biggest challenge in characterizing the SNP properly and effectively. Hence, the diversity/similarity profiles based on the Hill numbers are ideal for dealing with the challenge from the non-random distribution nature of human SNPs mentioned previously. The diversity profile also avoids a serious issue associated with most existing diversity indexes, *i.e*., there was not a single diversity index that can comprehensively measure diversity but multiple indexes (such as Shannon and Simpson indexes) are not comparable with each other. This makes the choice of diversity index often confusing for practitioners: which one, Simpson’s index or Shannon’s index is better?

Before proceeding to propose and develop our individual-level SNP diversity, here we summarize the following four points to answer a possible question from readers. Why bother to introduce another level of diversity even if it can be properly defined? (*i*) The SNP *alpha*- diversity profile offers a series of metrics for characterizing the SNP patterns of an individual genome, which is personal and individual-specific at the whole genome level. (*ii*) It also offers a cross-scale tool for comparing individuals and complements the population level analysis. For example, the SNP *beta*-diversity (we propose) is defined to compare two or more individuals within a population in their SNP distribution variation patterns. The SNP *gamma*-diversity (we propose) is defined to measure the total diversity (variations) of all individuals within a population. (*iii*) The study also presents another example of the cross-fertilizing between population genetics and community ecology. (*iv*) In our opinion, the case for developing an *individual-level* genetic diversity is particularly compelling in the genomics era when the genetic information of an individual in the form of DNA sequences is readily available, while in the 1970s, the data for individual-level is much smaller and only when population data became big enough were formal metrics required. As a side note, our proposed metrics can also be applied to extend *population-level* genetic diversity, which we will address in a follow-up study.

## Concepts and Definitions

Let us start with a brief review on the species diversity (*aka* community diversity, biodiversity or ecological diversity) to explain the two essential elements of diversity concept in general, which should facilitate the introduction of our SNP diversity and similarity measures below. Species diversity refers to the ecological diversity of species in an ecological community, but diversity concept is equally applicable to genetic diversity (*e.g*. Nei 1973, Wehenkel *et al*., Bergmann *et al*.)^13,23,24^ or other entities such as metagenome diversity (Ma and Li)^20^. Conceptually, diversity possesses two essential elements: the *variety* and the *variability* of *varieties*; (Gaston; Chao *et al*.)^10,25^. For example, the two elements of species diversity are species (variety) and the variability of species abundances. To quantify the concept of species diversity, one surveys a community (usually by *sampling*), counts the abundances of each species in the community, and obtains *p*_i_ = (the relative abundance of species *i*) = (the number of individuals of species *i*)/(the total individuals of all species in the community), and also counts the number of species in the community (*S*). The dataset from such a survey (sampling) is a vector of species abundance in the form of (*p*_1_*, p*_2_*, …, p*_*i*_, *…p*_*s*_). For such a vector of relative abundances (frequencies), one approach to characterizing it is to fit a statistical distribution, which is known as species abundance distribution (SAD) in community ecology. The most widely used SADs include log-series, log-normal, and power law distributions; a common property of SADs is that they are highly skewed, long tail distributions, but rarely follow the normal distribution or uniform distribution. Instead, the SAD is highly aggregated (skewed or non-random), just as the non-random SNP distribution previously mentioned in the introduction section. Although SAD fully describes the species abundance frequency and therefore adequately captures the full characteristics of species diversity, using a SAD to measure diversity fails to present intuitive measures to synthesize the two elements of diversity (*i.e*., variety and variability). An alternative approach to fitting SAD is to use various diversity metrics (also known as measures or indexes). Numerous diversity metrics for measuring species diversity have been proposed, with Shannon’s entropy being the most well known.

Diversity metrics belong to the so-termed *aggregate* functions, which combine several values into a single value (Beliakov *et al*., James)^6,7^. The arithmetic mean (average) is the most commonly utilized aggregation function, but it is a rather poor metric for measuring diversity due to the highly non-random distribution of species abundances. Instead, entropy-based aggregation function is suitable for measuring diversity. The first and also still one of the most widely utilized entropy-based diversity metric is Shannon entropy, which was attributed to Claude Shannon, the co-founder of information theory; (Shannon, Shannon & Weaver)^8,26^, but Shannon had never studied biodiversity himself. What happened was that ecologists borrowed the idea from Shannon’s information theory, in which Shannon’s entropy measures the content of information or uncertainty in communication systems. Of course, Shannon’s entropy is indeed sufficiently general for measuring biodiversity because diversity is essentially heterogeneity, and heterogeneity and uncertainty both can be measured by the change of information, *i.e*., information lowers uncertainty.

Using Shannon entropy as example, species diversity (*H*), more accurately species evenness, can be computed with the following formula,

1 $$H=\,-{\sum }_{i=1}^{S}{p}_{i}\,\mathrm{ln}({p}_{i})$$

where *S* is the number of species in the community, and *p*_i_ is the relative abundance of each species in the community. In terms of the *variety-variability* notion for defining diversity, the *variety* is the species and *variability* is the species abundance obviously. In fact, the variety-variability notion can be utilized to define diversity for any systems (not even limited to biological systems) that can be abstracted as the two elements of variety and variability, including SNP diversity, as exposed below.

### Definitions for SNP diversities

**Figure 1:**
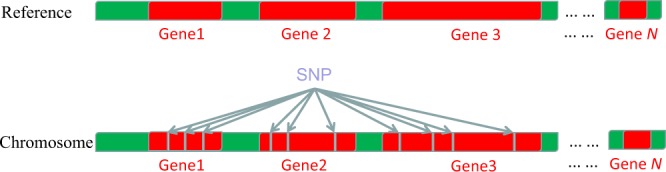
A conceptual diagram showing the distribution of SNPs on a chromosome with reference to the reference chromosome: the chromosome is similar to an ecological community, and the number of SNPs on a gene locus is similar to the species abundance in an ecological community. For example, there are three SNPs on the locus of gene-1, assuming the total SNPs on the chromosome is *N* (or 10 displayed with the first 3 genes displayed), then the relative SNP abundance for gene-1 is equal to 3/*N* (or 3/10 = 0.3 with the 3 genes displayed). Similarly, *p*_2_, *p*_3_, … can be computed. When the relative abundances of SNPs are available, the diversity (Hill numbers) can be computed based on the diversity definitions [Eqs. (2–15)]. The R-codes computing alpha-diversity, beta-diversity (including similarity) profiles are provided in the OSI.

Using an analogy, a chromosome that has many *loci* is similar to an ecological community of many species, and each *locus* may have different number of SNPs. With variety-variability notion for defining diversity, the locus is the *variety* (similar to species in a community), and the number of SNPs at each locus is the *variability* (similar to species abundance in a community). Assuming *S* is the number of *loci* with any SNP, and *p*_i_ is the *relative* abundance of SNPs at locus *i* (*i.e*., the number or abundance of SNPs at locus *i* divided by the total number of SNPs from all loci), then SNP diversity can be measured with Shannon entropy (Eq. 1). Strictly speaking, SNP may also be termed *locus* diversity, since *locus* is essentially the ‘habitat’ where SNPs reside. Figure 1 conceptually illustrated the distribution of SNPs on a chromosome; specifically how *p*_*i*_ is defined and computed.

Although Shannon’s entropy has been widely used for measuring species diversity, a recent consensus among ecologists is that Hill numbers, which are based on Renyi’s general entropy, offer the most appropriate metrics for measuring alpha-diversity and for multiplicatively partitioning beta-diversity (Chao *et al*. 2012, 2014, Ellison 2010, Kaplinsky & Arnaout)^9,10,12,19^. Given the advantages of Hill numbers over other existing diversity indexes, we believe that the Hill numbers should also be a preferred choice for defining the SNP diversity.

### SNP alpha-diversity

Hill numbers were derived by Hill (1973) based on Renyi’s (1961) general entropy^15,16^. Here we propose to apply it for defining the SNP alpha-diversity, *i.e*.,

2 $${}^{q}D={(\mathop{\sum }\limits_{i=1}^{G}{p}_{i}^{q})}^{1/(1-q)},$$

where *G* is the number of gene loci with any SNP, *p*_i_ is the relative abundance (*i.e*., the *frequency of occurrence*) of SNPs at locus *i*, *q* = 0, 1, 2, … is the *order* number of SNP diversity, ^*q*^*D* is the SNP alpha-diversity at diversity order *q*, *i.e*., the Hill numbers of the *q*-th order.

The Hill number is undefined for *q* = 1, but its limit as *q* approaches to 1 exists in the following form:

3 $${}^{1}D=\mathop{\mathrm{lim}}\limits_{q\to 1}{}^{q}D=\exp (-\mathop{\sum }\limits_{i=1}^{G}{p}_{i}\,\log ({p}_{i}))$$

The diversity order (*q*) determines the sensitivity of the Hill number to the relative abundance (*i.e*., the frequency of occurrence) of SNP. When *q* = 0, the SNP frequency does not count at all and ^0^*D* = *G, i.e*., the *SNP richness*, similar to the *species richness* in species diversity concept. When *q* = 1, ^1^*D* equals the *exponential* of Shannon entropy, and is interpreted as the number of SNPs with typical or common frequencies. Hence, Shannon index is essentially a special case of Hill numbers at diversity order *q* = 1. When *q* = 2, ^2^*D* equals the reciprocal of Simpson index, *i.e*.,

4 $${}^{2}D=(1/\mathop{\sum }\limits_{i=1}^{G}{p}_{i}^{2})$$

which is interpreted as the number of dominant or very frequently occurred SNPs. Therefore, two most widely used diversity indexes, Shannon index and Simpson index are the special cases, and more accurately, the functions of the Hill numbers.

In general, we need to specify an entity (unit or scope) for defining and measuring SNP diversity. For demonstrative purpose in this article, we choose individual chromosome as the entity for defining SNP diversity, similar to using community for defining species diversity. The general interpretation of diversity of order *q* is that the chromosome contains ^*q*^*D* = *x* loci with equal SNP frequency. Note that the entity for defining SNP diversity can be other appropriate units such as the *whole genome* of an organism or segment of chromosome.

The above-defined SNP diversity measures the diversity of SNP on an individual genetic entity (such as chromosome or genome), similar to the concept of alpha diversity in community species diversity, and we term it *SNP alpha-diversity*. In the following, we define the counterparts of species beta-diversity and gamma-diversity in community ecology for SNPs, *i.e*., *SNP beta-diversity* and *SNP gamma-diversity*.

### SNP gamma diversity

While the previously defined SNP alpha-diversity is aimed to measure the SNP diversity within a genetic entity (such as a chromosome or genome), the following SNP gamma-diversity is defined to measure the *total* SNP diversity of pooled, multiple (*N*) chromosomes from a population (cohort) of *N* different individuals, one from each individual but with the same chromosome numbering.

Assuming there are *N* individuals in a population (cohort), we define the *SNP gamma-diversity* with the following formula, similar to the species gamma-diversity in ecology (*e.g*., Chao *et al*.; Chiu *et al*.)^9,10,27^,

5 $${}^{q}D_{\gamma }={(\mathop{\sum }\limits_{i=1}^{G}{(\overline{{p}_{i}})}^{q})}^{1/(1-q)},$$

where $\overline{{p}_{i}}$ is the SNP frequency on the *i*-th locus (*i* = 1*, 2, …,G*) in the pooled population of *N* individuals (termed *N*-population).

Comparing Eq. (5) for gamma diversity with Eq. (2) for alpha diversity reveals that the gamma-diversity is the Hill numbers based on the SNP *frequency* at *i*-th locus in the *N*-population. Similar to Chao *et al*.^9,10^ Chiu *et al*.^27^, derivation for species gamma-diversity in ecological community, assuming *y*_*ij*_ is the SNP frequency at *i*-th locus of *j-*th individual, *y*_*i*+_ is the total value of SNP at *i*-th locus contained in the *N* individuals, *y*_+*j*_ is the total SNP from *j*-th individual, *y*_++_ is the total SNP contained in *N* individuals, *p*_*ij*_ is the SNP frequency at *i*-th locus of *j*-th individual, *w*_*j*_ is the weight of the *j*-th individual,

$$\begin{array}{rcl}{y}_{i+} & = & {\sum }_{j=1}^{N}{y}_{ij}=y{y}_{++}{\sum }_{j=1}^{N}{w}_{j}{p}_{ij}\\ {y}_{+j} & = & {\sum }_{i=1}^{G}{y}_{ij}\\ {y}_{++} & = & {\sum }_{i=1}^{G}{\sum }_{j=1}^{N}{y}_{ij}\\ {p}_{ij} & = & {y}_{ij}/{y}_{+j}\\ {w}_{j} & = & {y}_{+j}/{y}_{++},\,{\sum }_{j=1}^{N}{w}_{j}=1,\end{array}$$

it can be easily derived that,

6 $$\overline{{p}_{i}}=({y}_{i+}/{y}_{++})={\sum }_{j}^{N}({w}_{j}{p}_{ij}).$$

Plug Eq. (6) for $\overline{{p}_{i}}$ into the definition of *SNP gamma diversity* [Eq. (5)], we obtain the following formulae for computing *SNP gamma-diversity* of *N*-population as follows:

7 $${}^{q}D_{\gamma }={(\mathop{\sum }\limits_{i=1}^{G}{(\overline{{p}_{i}})}^{q})}^{1/(1-q)}={\left\{\mathop{\sum }\limits_{i=1}^{G}{\left(\frac{{y}_{i+}}{{y}_{++}}\right)}^{q}\right\}}^{1/(1-q)}={\{\mathop{\sum }\limits_{i=1}^{G}{(\mathop{\sum }\limits_{j=1}^{N}{w}_{j}{p}_{ij})}^{q}\}}^{1/(1-q)}(q\ne 1)$$

8 $${}^{1}D_{\gamma }=\mathop{\mathrm{lim}}\limits_{q\to 1}{}^{q}D_{\gamma }=\exp \left\{-\mathop{\sum }\limits_{i=1}^{G}\left(\frac{{y}_{i+}}{{y}_{++}}\right)\log \left(\frac{{y}_{i+}}{{y}_{++}}\right)\right\}=\exp \{-\mathop{\sum }\limits_{i=1}^{G}(\mathop{\sum }\limits_{j=1}^{N}{w}_{j}{p}_{ij})\log (\mathop{\sum }\limits_{j=1}^{N}{w}_{j}{p}_{ij})\}\,(q=1)$$

### SNP beta diversity

In community ecology, there are two schemes for defining beta-diversity: one is the additive partition and another is the multiplicative partition of gamma diversity into assumingly independent alpha-diversity and beta-diversity. Recent consensus (*e.g*., Jost; Ellison; Chao *et al*., Gotelli & Chao, Gotelli & Ellison)^9–12,28,29^ recommended the use of multiplicative partition. Let (${}^{q}D_{\alpha }$) and (${}^{q}D_{\gamma }$) are alpha- and gamma-diversity measured with the Hill numbers, respectively, beta-diversity is defined as:

9 $${}^{q}D_{\beta }={}^{q}D_{\gamma }/{}^{q}D_{\alpha }$$

We adopt the exactly same multiplicative partition of the Hill numbers in species diversity for measuring SNP beta-diversity except that both alpha- and gamma- diversities are computed with SNP frequency (relative abundance), rather than with species abundances.

This SNP beta-diversity (${}^{q}D_{\beta }$) derived from the above multiplicative partition takes the value of 1 if all communities are identical, and the value of *N* (the number of individuals in the population) when all individuals are completely different from each other (*i.e*., no shared SNPs).

Although Eq. (2) correctly defines the SNP alpha-diversity, it requires some adaptations to apply for the partition of gamma diversity in order to obtain beta-diversity with Eq. (9). Similar to the derivation for species alpha diversity as demonstrated in Chiu *et al*.^27^, we can derive the following formulae for SNP alpha diversity in *N*-population setting, *i.e*.,

10 $${}^{q}D_{\alpha }=\frac{1}{N}{\left\{\mathop{\sum }\limits_{i=1}^{G}{\mathop{\sum }\limits_{j=1}^{N}\left(\frac{{y}_{i+}}{{y}_{++}}\right)}^{q}\right\}}^{1/(1-q)}=\frac{1}{N}{\{\mathop{\sum }\limits_{i=1}^{G}\mathop{\sum }\limits_{j=1}^{N}{({w}_{j}{p}_{ij})}^{q}\}}^{1/(1-q)}(q\ne 1)$$

11 $$\begin{array}{c}{}^{1}D_{\alpha }=\mathop{\mathrm{lim}}\limits_{q\to 1}{}^{q}D_{\gamma }=\exp \left\{-\mathop{\sum }\limits_{i=1}^{G}\sum \left(\frac{{y}_{ij}}{{y}_{++}}\right)\log \left(\frac{{y}_{ij}}{{y}_{++}}\right)-\,\log (N)\right\}\\ \,=\,\exp \{-\mathop{\sum }\limits_{i=1}^{G}\mathop{\sum }\limits_{j=1}^{N}({w}_{j}{p}_{ij})\log ({w}_{i}{p}_{ij})-\,\log (N)\}\,(q=1)\end{array}$$

The computation of SNP beta-diversity can then be accomplished with Eqs. (7–11), *i.e*., Eqs. (7 and 8) for gamma diversity, (9) for beta-diversity and (10–11) for alpha-diversity.

We define a series of the Hill numbers for SNP diversity at different diversity order *q* = 0, 1, 2, … as *SNP diversity profile*, that is, a series of Hill numbers corresponding to different non-linearity levels weighted differently with the SNP frequency distribution.

## The Definitions for SNP Similarities

Similar to previous definition for the SNP diversity based on the Hill numbers, we can also define Hill-numbers-based similarity measures for comparing SNP similarities. We adopted the same mathematical formulae previously used for defining the community similarity measures (profiles) by Chao *et al*. and Chiu *et al*.^9,10,27^. In community ecology Chiu *et al*.^27^ showed that the four existing similarity measures, Jaccard, Sørensen, Horn, Morisita-Horn are actually functions of the beta diversity (${}^{q}D_{\beta }$) measured in the Hill numbers. Similar to the previously defined diversity profile, the four similarity indexes we define below form a series of *SNP similarity profile*. In the following, we define the four similarity measures in the context of *N*-populations of individuals. A major benefit of using these similarity measures, rather than the beta-diversity directly, is that the similarity indexes are ‘normalized’ to the range of [0, 1] by the number of individuals (*N*). If the beta-diversity is directly used to compare the similarity, the beta-diversity of *N*-population ranges from 1 to *N*, which make the comparisons being dependent on the number of individuals (*N*).

### Local SNP overlap (C_qN_)

The *local SNP overlap* measure (*C*_*qN*_) quantifies the effective average proportion of SNPs that are shared across all *N* individuals:

12 $${C}_{qN}=\frac{{(1/{}^{q}D_{\beta })}^{q-1}-{(1/N)}^{q-1}}{1-{(1/N)}^{q-1}}$$

where ${}^{q}D_{\beta }$ is the SNP beta-diversity at order *q* computed with Eq. (8), *N* is the number of individuals in the population. When *q* = 0, *C*_*qN*_ is actually the Sørensen similarity index; *q* = 1, *C*_*qN*_ is the Horn similarity index; *q* = 2, *C*_*qN*_ is the Morisita-Horn similarity index.

### Regional SNP overlap (UqN)

The *regional SNP overlap* measure (*U*_*qN*_) quantifies the effective proportion of shared SNPs in the pooled *N*-population:

13 $${U}_{qN}=\frac{{(1/{}^{q}D_{\beta })}^{1-q}-{(1/N)}^{1-q}}{1-{(1/N)}^{1-q}}$$

when *q *= 0, this statistic is equivalent to Jaccard similarity measure; *q *= 1, it is equivalent to Horn similarity; *q *= 2, it is equivalent to Morisita-Horn similarity index.

### SNP homogeneity measures (S_qN_)

*S*_*qN*_ quantifies the *SNP homogeneity* (evenness) in an N-population:

14 $${S}_{qN}=\frac{1/{}^{q}D_{\beta }-1/N}{1-1/N}$$

when *q *= 0, this statistic is equivalent to Jaccard similarity measure; *q *= 2, it is equivalent to Morisita-Horn similarity index.

### SNP turnover complement (V_qN_)

The complement of *V*_*qN*_ linearly quantifies the relative SNP turnover rate per individual. It represents the proportion of a typical individual that changes from one individual to another individual.

15 $${V}_{qN}=\frac{N-{}^{q}D_{\beta }}{N-1}=1-\frac{{}^{q}D_{\beta }-1}{N-1}$$

when *q *= 0, this statistic is equivalent to Sørensen similarity measure; *q *= 2, it is equivalent to Morisita-Horn similarity index.

## Demonstration with 1000-Genomes Project

### The datasets for the demonstration

We used the SNP datasets obtained through the whole genome sequencing data from 1000-Genomes Project^3,22^. Through a series of bioinformatics analyses, the list of all loci with SNP mutations, and the number of loci with SNP mutations on each chromosome were obtained from the raw sequence reads. Detailed information on sequencing and bioinformatics procedures for obtaining the SNP datasets from the whole-genome sequencing of the DNA samples is referred to 1000-Genomes Project^3,22^. A total of 2504 individuals were sampled from 5 populations: Africa (AFR), Americas (AMR), Europe (EUR), East Asia (EAS) and South Asia (SAS). They characterized in total over 88 million variants (84.7 million single nucleotide polymorphisms (SNPs), 3.6 million short insertions/deletions (indels), and 60,000 structural variants), all phased onto high-quality haplotypes^3,22^.

The R-codes for computing alpha-diversity, beta-diversity (including similarity) profiles are provided in the online supplementary information (OSI).

### Demonstrations of the SNP Alpha-Diversity

#### Chromosome level SNP alpha-diversity profile

Table S1 in the OSI (online supplementary information: Excel file) listed the SNP alpha-diversity on each chromosome of each individual from each population (ethnic group) in the 1000-Genomes Project. It contained very basic SNP alpha-diversity for each individual’s each chromosome at each diversity order *q* = 0–4, according to previous formulae for computing alpha-diversity.

**Figure 2:**
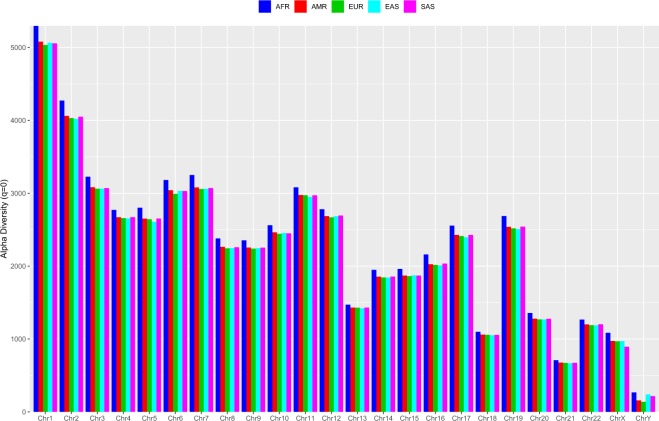
The mean (per individual) SNP alpha-diversity (*q* = 0) at the chromosome level for the 1000-Genomes Project: SNP (alpha) diversity at order *q* = 0 measures the number of loci with any number of SNPs, *i.e*., SNP *richness* (for which the SNP abundance does not weigh).

Table S2 summarized the average SNP diversity (per individual within a population or ethnic group) on each chromosome of each population from Table S1, *i.e*., averaged across all individuals within a population. Table 1 (the top section) below is excerpted partial results from Table S2 in the OSI to facilitate illustration. The *SNP diversity profile* (the Hill numbers at different diversity orders) offers an effective tool to assess different mutation profiles on different chromosomes, different individuals in a population, or different populations of a species. Figure 2 illustrates the average SNP diversity on each chromosome for each population, for diversity order *q* = 0. The graphs for diversity order *q* = 1–4 are included in Figs. S1–S4 of the OSI.

**Table 1:** The mean (*per individual* within a population) SNP alpha-diversity at the chromosome level (*i.e*., on each chromosome), averaged across all individuals in same population, excerpted from Table S2, which, in turn, was summarized from Table S1 for the alpha-diversity of each individual on each chromosome in the 1000-Genomes Project with five ethnic groups including African (AFR), American (AMR), European (EUR), East Asian (EAS) and South Asian (SAS).

Chromosomes	Populations	*q* = 0	*q* = 1	*q* = 2	*q* = 3	*q* = 4
Chr1	AFR	5296.0	1594.630	727.223	441.365	322.514
	AMR	5080.6	1513.867	694.462	424.723	311.736
	EUR	5035.5	1505.179	693.988	427.136	315.301
	EAS	5064.8	1520.801	696.132	421.306	306.225
	SAS	5057.3	1504.909	684.730	415.486	304.005
… …	… …	… …	… …	… …	… …	… …
ChrY	AFR	266.5	136.715	63.669	38.270	28.807
	AMR	157.3	87.454	44.957	28.818	22.379
	EUR	137.2	77.786	40.685	26.077	20.218
	EAS	238.7	136.654	71.336	44.499	33.626
	SAS	215.3	125.332	67.853	44.271	34.494
Mean (per Chromosome) for each Population	AFR	2355.1	665.344	297.707	181.709	134.417
	AMR	2242.0	632.056	283.999	174.548	129.510
	EUR	2226.1	628.972	284.031	174.981	129.898
	EAS	2229.8	630.901	283.501	173.976	128.980
	SAS	2238.2	633.261	284.884	174.948	129.701
*The following is summarized from Table S3 containing the *p*-values of Wilcoxon tests for the difference in the alpha-diversity between each pair of ethnic groups (populations)						
	**Comparison**	***q* = 0**	***q* = 1**	***q* = 2**	***q* = 3**	***q* = 4**
Percentage (%) with significant differences in the SNP-alpha diversity (at the chromosome level) between pair-wise ethnic groups	AFR vs. AMR	100.0	100.0	87.5	83.3	79.2
	AFR vs. EUR	100.0	100.0	91.7	95.8	87.5
	AFR vs. EAS	100.0	100.0	95.8	91.7	91.7
	AFR vs. SAS	100.0	100.0	91.7	83.3	83.3
	AMR vs. EUR	95.8	66.7	58.3	70.8	62.5
	AMR vs. EAS	87.5	75.0	91.7	91.7	83.3
	AMR vs. SAS	62.5	58.3	66.7	66.7	66.7
	EUR vs. EAS	87.5	79.2	70.8	83.3	87.5
	EUR vs. SAS	87.5	79.2	62.5	75.0	79.2
	EAS vs. SAS	91.7	70.8	79.2	75.0	83.3

*Summarized from Table S3: The *p*-value from Wilcoxon tests for the SNP alpha-diversity between different ethnic groups (populations).

We further compared the SNP alpha-diversity at the chromosome level among five different populations with Wilcoxon tests (Table S3). The bottom section of Table 1 excerpted the summary test results from Table S3 in the OSI. It is shown that extensive differences (58.3–100%) exist among five different populations, and the variability (level of differences) depends on population and diversity order (*see* bottom section of Table 1, and Table S3 for the details). This demonstrates the power of the Hill numbers in detecting the SNP variability at the chromosome level among different populations (ethnic groups).

The previous results of the SNP alpha-diversity at the chromosome level demonstrate at least the following three implications. First, it provides a series of diversity metrics (*i.e*., the diversity profile) to characterize the mutation profile of an individual’s specific chromosome in comparison with the reference genome. This chromosome-level diversity profile is both individual and chromosome specific. If time-series data (*e.g*., medical records including periodic

sequencing of an individual’s genome) of the diversity profile for an individual are available, then the dynamics of the diversity profile can provide potentially valuable information on the personalized disease-risk assessment and prediction for the individual. Second, the diversity profile can also be applied to compare the variation patterns of the SNP between two populations (cohorts) as demonstrated previously. Third, the ‘unit’ for measuring diversity can be other than chromosome, for examples, a segment of a chromosome, or even a gene cluster of specific function(s) (*e.g*., specific diseases).

#### Genome level SNP alpha-diversity profile

**Figure 3:**
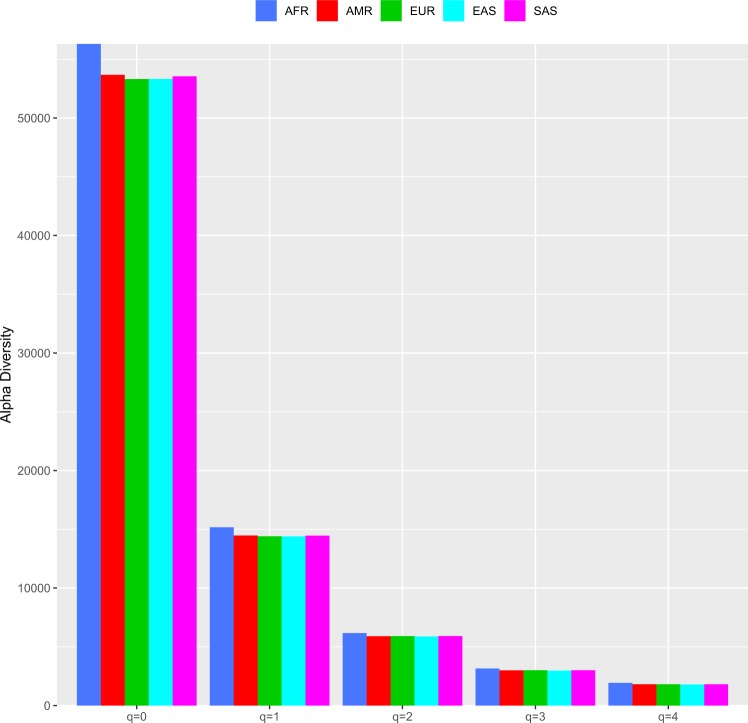
The mean SNP alpha-diversity at genome level for each diversity order (*q* = 0–4) for the five populations of the 1000-Genomes Project.

Table S4 in the OSI (Excel file) listed the SNP alpha-diversity of each individual (*i.e*., at the whole genome level and computed by combining the SNPs from all chromosomes of an individual’s genome) from each population (ethnic group) in the 1000-Genomes Project. Table 2 (the top section) below summarized the average SNP alpha diversity (per individual) for each population from Table S4 in the OSI. Figure 3 illustrated the average SNP alpha diversity at the genome level for each population, for each diversity order from *q* = 0 to 4. We further compared the SNP alpha-diversity at the whole genome level among five different populations with Wilcoxon tests (Table S5). The bottom section of Table 2 (which is summarized from Table S5) shows that extensive differences (70–90%) exist among five different populations, and the variability level depends on population and/or diversity order. At lower diversity order, the percentages were higher (90% for *q* = 0, and 80% for *q* = 1), and the percentages were lower at high diversity orders (70% for *q* = 2–4). This result demonstrates the power of the Hill numbers in measuring SNP diversity and discerning the variability at the whole-genome level.

**Table 2:** The mean SNP alpha-diversity at genome level (including all his or her chromosomes) averaged across the all individual in same population (summarized from Table S4 for the alpha-diversity at genome level in the 1000-Genomes Project).

Populations		*q* = 0	*q* = 1	*q* = 2	*q* = 3	*q* = 4
AFR	Mean	**56306.8**	**15167.778**	**6165.103**	**3150.498**	**1928.773**
	Std. Err.	**36.5**	**8.351**	**3.501**	**2.524**	**2.050**
AMR	Mean	**53678.6**	**14461.986**	**5898.441**	**2993.577**	**1813.559**
	Std. Err.	**53.3**	**12.357**	**5.677**	**4.671**	**3.989**
EUR	Mean	**53311.0**	**14403.534**	**5906.719**	**3000.368**	**1812.331**
	Std. Err.	**37.5**	**8.616**	**3.892**	**3.023**	**2.532**
EAS	Mean	**53318.6**	**14392.431**	**5870.595**	**2968.880**	**1792.056**
	Std. Err.	**37.4**	**8.425**	**3.842**	**3.200**	**2.747**
SAS	Mean	**53542.3**	**14458.647**	**5903.536**	**2994.627**	**1812.465**
	Std. Err.	**38.1**	**8.860**	**4.089**	**3.245**	**2.708**
*The following is summarized from Table S5 containing the *p*-values of Wilcoxon tests for the differences in the alpha-diversity of the whole genome between each pair of populations						
**Percentage (%) of pairs (of populations) with significant differences**		***q*** = **0**	***q*** = **1**	***q*** = **2**	***q*** = **3**	***q*** = **4**
		90	80	70	70	70

*Summarized from Table S5: The p-value of Wilcoxon tests for the SNP-alpha diversity of the whole genome among different ethnic groups (populations).

The genome-level SNP alpha diversity profile possesses similar implications as the previous chromosome-level profile. For example, the dynamics of genome-level diversity profiles may offer personalized medicine insights for individuals, as well as epidemiology insights when multiple cohorts are compared. In addition, it also offers a simple but powerful approach to compare the mutations patterns of the genomes from different individuals, or from different ethnic groups.

### Demonstrations of the SNP Beta-Diversity and similarities

#### Chromosome level SNP beta-diversity profile

We demonstrate the computation of *SNP beta-diversity* with a slightly different scheme from the computation of SNP alpha-diversity. That is, we compute the pair-wise SNP beta-diversity and similarity for the same (numbered) chromosome between any two individuals in the 1000-Genomes cohort. To reduce computational load but still obtain representative results, we randomly sampled 100 individuals from each of the five populations, and the SNP data of 500 individuals in total were used to compute the pair-wise SNP beta-diversity. We computed the averages of the SNP beta-diversity or similarity values of all the pairs sampled (a total of 10,000 pairs for each chromosome for each pair of populations), and reported the mean beta-diversity and similarity on each chromosome for pairs of populations (Table S6). Interestingly, the sex chromosomes exhibited the highest beta-diversity values or lowest similarity values between different populations. Since beta-diversity is defined and computed in pair-wise manner, further statistical significance tests for comparing the pairs are of rather limited biomedical meaning and were omitted.

#### Genome level SNP beta-diversity profile

Similar to previous genome level SNP alpha-diversity, we also computed the genome level SNP beta-diversity. We again randomly sampled 100 individuals from each of the five populations, and pooled together the SNPs from all chromosomes of an individual to compute pair-wise genome-level beta-diversity between two individuals from two respective populations. A total of 10,000 pairs of the beta-diversity for each pair of populations (e.g., AFR vs. AMR) were computed, and the average of the 10,000 beta-diversity values were displayed in Table 3. These values of beta-diversity and similarity are as expected, *e.g*., the beta-diversity of two individuals ranged between 1 and 2, and all the similarity values normalized between 0 and 1. Similar to the previous chromosome level beta-diversity, statistical tests for comparing the differences among populations were omitted because of their limited biomedical meaning.

**Table 3:** The means of pair-wise genome-level SNP beta-diversity and similarity measures between any two individuals from their respective populations.

Chromosome	*q* = 0					*q* = 1					*q* = 2				
	*Beta*	Four Similarity Measures				*Beta*	Four Similarity Measures				*Beta*	Four Similarity Measures			
		*C*_*q*_	*U*_*q*_	*S*_*q*_	*V*_*q*_		*C*_*q*_	*U*_*q*_	*S*_*q*_	*V*_*q*_		*C*_*q*_	*U*_*q*_	*S*_*q*_	*V*_*q*_
AFR vs. AMR	1.079	0.921	0.854	0.854	0.921	1.038	0.946	0.946	0.926	0.962	1.026	0.949	0.974	0.949	0.974
AFR vs. EUR	1.081	0.919	0.851	0.851	0.919	1.040	0.943	0.943	0.922	0.960	1.028	0.945	0.972	0.945	0.972
AFR vs. EAS	1.081	0.919	0.850	0.850	0.919	1.040	0.943	0.943	0.923	0.960	1.028	0.946	0.972	0.946	0.972
AFR vs. SAS	1.079	0.921	0.854	0.854	0.921	1.038	0.946	0.946	0.927	0.962	1.026	0.949	0.974	0.949	0.974
AMR vs. EUR	1.075	0.925	0.861	0.861	0.925	1.035	0.950	0.950	0.932	0.965	1.020	0.960	0.980	0.960	0.980
AMR vs. EAS	1.075	0.925	0.860	0.860	0.925	1.035	0.950	0.950	0.932	0.965	1.020	0.960	0.980	0.960	0.980
AMR vs. SAS	1.075	0.925	0.861	0.861	0.925	1.035	0.951	0.951	0.933	0.965	1.020	0.961	0.980	0.961	0.980
EUR vs. EAS	1.078	0.922	0.855	0.855	0.922	1.037	0.948	0.948	0.929	0.963	1.021	0.959	0.979	0.959	0.979
EUR vs. SAS	1.075	0.925	0.861	0.861	0.925	1.035	0.951	0.951	0.933	0.965	1.020	0.961	0.980	0.961	0.980
EAS vs. SAS	1.075	0.925	0.861	0.861	0.925	1.034	0.951	0.951	0.933	0.966	1.020	0.962	0.980	0.962	0.980
Mean	1.077	0.923	0.857	0.857	0.923	1.037	0.948	0.948	0.929	0.963	1.023	0.955	0.977	0.955	0.977
Std. Err.	0.001	0.001	0.001	0.001	0.001	0.001	0.001	0.001	0.001	0.001	0.001	0.002	0.001	0.002	0.001

The differences between genetic (SNP) alpha-diversity and beta-diversity are similar with those in community ecology. The latter provides a mean to quantify the differences between two or more individuals, either at chromosome, genome, or even population levels. The similarity profile is simply a more convenient recasting of beta-diversity for comparing different entities (chromosomes, genomes, or populations).

### Summary

SNPs may occur in coding sequences of genes, non-coding regions of genes, or in the inter-genic regions. Accordingly, the SNP diversity defined in this article can be applied separately to the three types of SNP occurrence regions. For demonstrative purpose, we did not distinguish the three types in this article, but all the definitions and computational procedures presented in previous sections can be directly applied to separate measuring of the SNP diversities. The only, but minor, difference would be in the data preparation step, *i.e*., the preparatory calculation of *p*_*i*_ according to the region chosen, either coding, non-coding, inter-genic, or the whole locus.

We demonstrated the SNP alpha-diversity with single chromosome and whole genome as the basic genetic entity for defining the genetic alpha-diversity, respectively, corresponding to the chromosome-level SNP and genome-level SNP alpha-diversity. For beta-diversity, we computed the *pair-wise* SNP beta-diversity for the *same-numbered* two chromosomes from two respective individuals, at the chromosome and genome level respectively. In fact, SNP beta-diversity may be computed for multiple (*N*) individuals, as defined previously. Besides defining and demonstrating SNP diversities, we also defined and demonstrated four similarity measures, all of Unlike beta-diversity, the similarity measures are normalized to [0, 1] and not their ranges are not influenced by the number of entities compared.

As argued previously, defining diversity requires two essential elements: the variety and the variability of varieties; (Gaston, Chao *et al*.)^10,25^. In the *individual-level* genetic diversity defined in this study, the *variety* can be SNP, deletion, duplication, inversion, insertion, translocation, or other mutational types. The calculation of *variability of varieties* is limited to *individual*, which is demonstrated with individual chromosome or genome in this article, but can also be region of chromosomes or group of loci, which may be particularly interested in by investigators. To calculate the *variability of varieties*, a reference genome is usually required, but the calculation does not require a population with more than two individuals. The latter is usually necessary for most existing definitions for the genetic diversity, which might be termed population-level genetic diversity to emphasize the distinction. We believe both types of genetic diversities have their own respective application domains and can even complement to each other.

The idea to use Renyi’s entropy^16^ for measuring ecological diversity originated more than a half century ago by Hill (1971), but his proposal received little attention until about a decade ago when a handful of ecologists (including Chao, Ellison, Jost etc) reintroduced the Hill numbers and achieved wide successes in community ecology^9–12,27,30^, which demonstrated the effectiveness and advantages of Hill numbers in assessing and interpreting ecological diversity. Recently Gaggiotti *et al*.^31^, developed a unifying framework for measuring biodiversity from genes to ecosystems by standardizing on the Hill number at diversity order *q* = *1*, which is a transformation of Shannon diversity index. Their simplification is necessary to develop a more generalized framework, but it does not obsolete the novelty of our work here. This is because, at a specific level (the genome level of an individual), Hill numbers at difference orders (*q* = 0, 1, 2, …) are still necessary to present a comprehensive diversity profile due to the complexity of the issues involved, as demonstrated in previous sections. Furthermore, as elaborated and demonstrated previously, a unique aspect of the present use of Hill numbers for measuring genetic diversity is that our definitions are at *individual* level, rather than at *population* level. To the best of our knowledge, this study introduces the first concept and definitions for the genetic diversity at individual level. The proposed concept and definitions should find new important applications in fields such as personalized precision medicine since they can be readily applied to monitor the change of individual-level mutations. Besides, the concept and metrics should also find novel applications in population genomics because the individual-level genomic metrics provide solid basic units for population-level analysis, which we will demonstrate in a follow-up study.

## Data availability

The SNP datasets from “1000-Genome Project” used in this study are publicly available:

https://www.internationalgenome.org.

## Supplementary information

Supplementary Information (I) PDF.

Supplementary Information (II) Excel Tables.
