# Supplementary material for: Defining Individual-Level Genetic Diversity and Similarity Profiles
Source: Sci Rep. 2020 Apr 2;10:5805. doi: 10.1038/s41598-020-62362-8 (PMC7118122; doi:10.1038/s41598-020-62362-8)
Supplement: Supplementary file 1 — Supplementary Information (I) PDF. [file 41598_2020_62362_MOESM1_ESM.pdf]

Online Supplementary Information (OSI) for: Ma ZS, Li LW and Zhang YP (2019) Defining Individual-Level Genetic Diversity and Similarity Profiles. *Scientific Reports*

List of Supplements

Tables S2, S3, S5, & S6. Tables S1 & S4 are included in Excel files

Supplementary Figures S1, S2, S3, S4

R-Codes for Computing Alpha-Diversity, Beta-Diversity and Similarity Metrics

Table S1. The SNP-alpha diversity on each chromosome of each individual in the 1000-Genomes Project (Excel file)

Table S2. The mean (*per individual* within a population) SNP alpha-diversity on each chromosome (at the chromosome level) averaged across all individuals in same population: summarized from Table S1 for the SNP alpha-diversity on each chromosome of each individual in the 1000-Genomes Project with five ethnic groups including African (AFR), American (AMR), European (EUR), East Asian (EAS) and South Asian (SAS)

**Chromosomes**	**Populations**	***q*=0**	***q*=1**	***q*=2**	***q*=3**	***q*=4**
Chr1	AFR	5296.0	1594.630	727.223	441.365	322.514
	AMR	5080.6	1513.867	694.462	424.723	311.736
	EUR	5035.5	1505.179	693.988	427.136	315.301
	EAS	5064.8	1520.801	696.132	421.306	306.225
	SAS	5057.3	1504.909	684.730	415.486	304.005
Chr2	AFR	4272.4	1177.721	533.779	327.393	241.935
	AMR	4061.3	1128.269	512.100	312.304	228.557
	EUR	4032.0	1122.050	509.032	306.302	220.254
	EAS	4020.9	1115.048	506.461	309.977	227.980
	SAS	4051.5	1125.352	510.612	310.431	226.277
Chr3	AFR	3226.4	853.189	392.716	251.388	190.692
	AMR	3082.4	823.893	378.615	240.065	180.767
	EUR	3062.2	817.491	375.955	238.730	179.953
	EAS	3062.9	823.747	383.862	246.727	187.514
	SAS	3070.1	824.662	380.009	240.928	181.075
Chr4	AFR	2771.7	799.163	383.177	245.351	186.577
	AMR	2670.2	784.563	380.555	243.096	182.390
	EUR	2658.9	782.129	377.793	239.757	178.714
	EAS	2653.9	782.664	378.795	242.183	182.706
	SAS	2672.1	782.376	377.140	240.041	180.029
Chr5	AFR	2800.7	850.552	446.119	303.384	238.204
	AMR	2651.8	808.863	425.955	291.025	228.849
	EUR	2642.8	805.494	426.092	293.527	232.247
	EAS	2607.8	791.762	415.878	283.279	222.314
	SAS	2653.5	811.364	428.554	293.167	230.476
Chr6	AFR	3182.1	918.023	448.378	270.401	188.881
	AMR	3041.0	880.579	437.194	270.204	191.323
	EUR	2992.0	865.928	432.077	267.974	190.347
	EAS	3033.2	875.631	429.599	261.371	182.923
	SAS	3030.2	882.757	438.785	270.264	190.932
Chr7	AFR	3250.8	754.542	294.365	164.909	115.291
	AMR	3080.7	724.446	288.168	162.803	114.399
	EUR	3057.5	724.419	291.739	165.536	116.281
	EAS	3061.1	725.540	291.404	166.521	118.123
	SAS	3072.1	726.102	290.393	164.919	116.202
Chr8	AFR	2380.0	549.574	180.124	83.937	55.411
	AMR	2264.6	512.036	158.917	73.054	48.443
	EUR	2244.2	507.978	157.173	72.031	47.730

	EAS	2246.3	510.106	157.890	71.663	47.337
	SAS	2258.3	511.949	159.798	73.326	48.545
Chr9	AFR	2353.8	619.606	225.550	106.915	70.033
	AMR	2255.4	592.654	213.993	103.335	68.755
	EUR	2239.9	593.456	216.971	105.455	70.224
	EAS	2244.8	595.476	216.072	105.287	70.469
	SAS	2253.7	594.678	215.811	104.630	69.653
Chr10	AFR	2561.4	662.738	274.963	156.902	110.931
	AMR	2463.0	646.309	273.928	158.076	111.906
	EUR	2443.0	646.179	279.103	163.273	116.363
	EAS	2453.3	647.375	267.664	149.519	103.866
	SAS	2449.6	646.948	276.644	159.778	112.851
Chr11	AFR	3082.0	734.311	291.120	178.190	135.772
	AMR	2976.3	719.524	293.355	181.869	138.637
	EUR	2972.7	721.191	294.932	182.870	139.347
	EAS	2948.3	711.433	289.021	178.821	136.092
	SAS	2972.1	715.971	289.908	178.846	135.951
Chr12	AFR	2781.1	887.489	446.156	292.722	223.695
	AMR	2685.6	842.080	421.309	276.896	211.540
	EUR	2670.1	836.614	418.390	274.784	210.170
	EAS	2684.5	852.528	431.018	284.692	217.993
	SAS	2693.9	847.571	426.478	281.462	215.417
Chr13	AFR	1471.0	409.478	201.936	133.488	102.852
	AMR	1431.7	404.771	201.675	134.679	104.751
	EUR	1429.6	405.339	203.286	136.929	107.234
	EAS	1420.6	398.279	195.804	129.320	99.964
	SAS	1430.7	399.487	197.351	131.029	101.481
Chr14	AFR	1948.8	486.623	194.800	111.378	81.093
	AMR	1855.5	465.893	186.858	107.393	78.671
	EUR	1844.7	468.110	189.621	107.947	78.310
	EAS	1843.2	463.040	185.001	105.529	76.802
	SAS	1857.0	466.146	187.067	106.607	77.470
Chr15	AFR	1961.2	554.270	276.276	184.260	142.292
	AMR	1871.0	529.416	262.536	174.622	134.868
	EUR	1861.8	527.346	261.121	173.301	133.771
	EAS	1872.7	530.645	263.914	176.312	136.492
	SAS	1870.2	529.622	260.876	172.202	132.262
Chr16	AFR	2159.9	442.518	99.847	45.382	31.432
	AMR	2025.5	401.427	91.010	41.855	29.164
	EUR	2015.8	396.872	90.834	42.060	29.349
	EAS	2009.7	395.067	88.274	40.283	28.038
	SAS	2034.9	403.787	90.566	41.583	28.989
Chr17	AFR	2555.1	808.022	381.283	245.468	187.547
	AMR	2428.2	770.298	364.717	235.471	180.388
	EUR	2412.1	764.247	362.937	235.282	180.663
	EAS	2396.3	749.111	350.419	224.704	171.025
	SAS	2427.4	766.874	361.629	233.128	178.179

Chr18	AFR	1098.1	334.052	177.111	122.991	96.987
	AMR	1059.8	326.886	177.050	125.391	100.555
	EUR	1057.0	325.277	177.354	126.243	101.459
	EAS	1051.3	328.023	179.060	127.286	102.165
	SAS	1056.5	326.583	178.301	127.023	102.162
Chr19	AFR	2687.1	1178.514	623.956	376.814	268.821
	AMR	2539.3	1068.645	555.975	338.345	243.697
	EUR	2518.1	1057.339	555.796	342.731	248.333
	EAS	2507.4	1048.921	545.547	333.945	241.746
	SAS	2542.3	1069.018	557.660	341.967	248.131
Chr20	AFR	1357.0	404.565	138.961	71.444	50.224
	AMR	1278.9	367.845	121.779	61.773	43.381
	EUR	1268.7	366.318	122.479	62.247	43.645
	EAS	1267.0	369.451	124.502	63.873	45.005
	SAS	1277.7	375.237	126.854	64.572	45.312
Chr21	AFR	708.9	216.306	110.600	74.085	57.505
	AMR	675.7	206.166	105.628	70.929	55.385
	EUR	672.0	205.903	104.521	69.394	53.714
	EAS	666.6	206.166	106.351	71.887	56.482
	SAS	673.2	207.353	106.176	71.221	55.669
Chr22	AFR	1266.4	350.378	139.021	81.734	60.498
	AMR	1199.8	338.002	137.539	81.341	60.078
	EUR	1189.0	340.085	141.272	84.836	63.239
	EAS	1187.9	338.123	141.761	84.951	62.631
	SAS	1202.2	340.819	139.125	81.958	60.237
ChrX	AFR	1085.4	245.275	93.847	52.847	38.011
	AMR	972.0	225.447	87.714	51.077	37.608
	EUR	968.7	232.590	93.603	55.128	40.696
	EAS	971.2	226.025	88.251	51.498	37.990
	SAS	894.8	213.367	84.890	49.923	37.017
ChrY	AFR	266.5	136.715	63.669	38.270	28.807
	AMR	157.3	87.454	44.957	28.818	22.379
	EUR	137.2	77.786	40.685	26.077	20.218
	EAS	238.7	136.654	71.336	44.499	33.626
	SAS	215.3	125.332	67.853	44.271	34.494
Means	AFR	2355.1	665.344	297.707	181.709	134.417
	AMR	2242.0	632.056	283.999	174.548	129.510
	EUR	2226.1	628.972	284.031	174.981	129.898
	EAS	2229.8	630.901	283.501	173.976	128.980
	SAS	2238.2	633.261	284.884	174.948	129.701

Table S3. The *p*-value from Wilcoxon tests for the SNP alpha-diversity between different populations (The bottom section listed the "Percentages (%) of Pairs with Significant Differences", *e.g.*, pair=AFR vs. AMR)

Chromosome	Treatments	*q*=0	*q*=1	*q*=2	*q*=3	*q*=4
Chr1	AFR vs. AMR	0.000	0.000	0.000	0.000	0.000
	AFR vs. EUR	0.000	0.000	0.000	0.000	0.000
	AFR vs. EAS	0.000	0.000	0.000	0.000	0.000
	AFR vs. SAS	0.000	0.000	0.000	0.000	0.000
	AMR vs. EUR	0.000	0.000	0.943	0.021	0.000
	AMR vs. EAS	0.000	0.000	0.126	0.005	0.000
	AMR vs. SAS	0.000	0.000	0.000	0.000	0.000
	EUR vs. EAS	0.000	0.000	0.066	0.000	0.000
	EUR vs. SAS	0.000	0.626	0.000	0.000	0.000
	EAS vs. SAS	0.000	0.000	0.000	0.000	0.009
Chr2	AFR vs. AMR	0.000	0.000	0.000	0.000	0.000
	AFR vs. EUR	0.000	0.000	0.000	0.000	0.000
	AFR vs. EAS	0.000	0.000	0.000	0.000	0.000
	AFR vs. SAS	0.000	0.000	0.000	0.000	0.000
	AMR vs. EUR	0.000	0.000	0.005	0.000	0.000
	AMR vs. EAS	0.000	0.000	0.000	0.010	0.712
	AMR vs. SAS	0.016	0.013	0.089	0.023	0.009
	EUR vs. EAS	0.000	0.000	0.001	0.000	0.000
	EUR vs. SAS	0.000	0.001	0.214	0.000	0.000
	EAS vs. SAS	0.000	0.000	0.000	0.953	0.005
Chr3	AFR vs. AMR	0.000	0.000	0.000	0.000	0.000
	AFR vs. EUR	0.000	0.000	0.000	0.000	0.000
	AFR vs. EAS	0.000	0.000	0.000	0.000	0.000
	AFR vs. SAS	0.000	0.000	0.000	0.000	0.000
	AMR vs. EUR	0.000	0.000	0.000	0.027	0.109
	AMR vs. EAS	0.000	0.930	0.000	0.000	0.000
	AMR vs. SAS	0.000	0.224	0.043	0.219	0.855
	EUR vs. EAS	0.710	0.000	0.000	0.000	0.000
	EUR vs. SAS	0.000	0.000	0.000	0.000	0.036
	EAS vs. SAS	0.000	0.105	0.000	0.000	0.000
Chr4	AFR vs. AMR	0.000	0.000	0.000	0.000	0.000
	AFR vs. EUR	0.000	0.000	0.000	0.000	0.000
	AFR vs. EAS	0.000	0.000	0.000	0.000	0.000
	AFR vs. SAS	0.000	0.000	0.000	0.000	0.000
	AMR vs. EUR	0.000	0.004	0.000	0.000	0.000
	AMR vs. EAS	0.000	0.037	0.010	0.092	0.483
	AMR vs. SAS	0.009	0.015	0.000	0.000	0.000
	EUR vs. EAS	0.000	0.277	0.129	0.000	0.000
	EUR vs. SAS	0.000	0.676	0.339	0.573	0.005
	EAS vs. SAS	0.000	0.631	0.018	0.001	0.000
Chr5	AFR vs. AMR	0.000	0.000	0.000	0.000	0.000
	AFR vs. EUR	0.000	0.000	0.000	0.000	0.000
	AFR vs. EAS	0.000	0.000	0.000	0.000	0.000

	AFR vs. SAS	0.000	0.000	0.000	0.000	0.000
	AMR vs. EUR	0.000	0.001	0.961	0.002	0.000
	AMR vs. EAS	0.000	0.000	0.000	0.000	0.000
	AMR vs. SAS	0.270	0.005	0.002	0.005	0.024
	EUR vs. EAS	0.000	0.000	0.000	0.000	0.000
	EUR vs. SAS	0.000	0.000	0.001	0.714	0.007
	EAS vs. SAS	0.000	0.000	0.000	0.000	0.000
Chr6	AFR vs. AMR	0.000	0.000	0.000	0.757	0.100
	AFR vs. EUR	0.000	0.000	0.000	0.040	0.181
	AFR vs. EAS	0.000	0.000	0.000	0.000	0.000
	AFR vs. SAS	0.000	0.000	0.000	0.797	0.174
	AMR vs. EUR	0.000	0.000	0.000	0.164	0.617
	AMR vs. EAS	0.002	0.000	0.000	0.000	0.000
	AMR vs. SAS	0.000	0.067	0.118	0.959	0.778
	EUR vs. EAS	0.000	0.000	0.025	0.000	0.000
	EUR vs. SAS	0.000	0.000	0.000	0.122	0.840
	EAS vs. SAS	0.430	0.000	0.000	0.000	0.000
Chr7	AFR vs. AMR	0.000	0.000	0.000	0.002	0.068
	AFR vs. EUR	0.000	0.000	0.000	0.324	0.080
	AFR vs. EAS	0.000	0.000	0.000	0.000	0.000
	AFR vs. SAS	0.000	0.000	0.000	0.713	0.139
	AMR vs. EUR	0.000	0.871	0.000	0.001	0.006
	AMR vs. EAS	0.000	0.233	0.000	0.000	0.000
	AMR vs. SAS	0.011	0.064	0.009	0.008	0.009
	EUR vs. EAS	0.044	0.113	0.757	0.038	0.000
	EUR vs. SAS	0.000	0.018	0.060	0.246	0.755
	EAS vs. SAS	0.000	0.380	0.090	0.000	0.000
Chr8	AFR vs. AMR	0.000	0.000	0.000	0.000	0.000
	AFR vs. EUR	0.000	0.000	0.000	0.000	0.000
	AFR vs. EAS	0.000	0.000	0.000	0.000	0.000
	AFR vs. SAS	0.000	0.000	0.000	0.000	0.000
	AMR vs. EUR	0.000	0.000	0.016	0.038	0.033
	AMR vs. EAS	0.000	0.036	0.259	0.002	0.000
	AMR vs. SAS	0.007	0.734	0.035	0.117	0.211
	EUR vs. EAS	0.047	0.000	0.092	0.154	0.024
	EUR vs. SAS	0.000	0.000	0.000	0.000	0.000
	EAS vs. SAS	0.000	0.001	0.000	0.000	0.000
Chr9	AFR vs. AMR	0.000	0.000	0.000	0.000	0.000
	AFR vs. EUR	0.000	0.000	0.000	0.000	0.825
	AFR vs. EAS	0.000	0.000	0.000	0.000	0.443
	AFR vs. SAS	0.000	0.000	0.000	0.000	0.047
	AMR vs. EUR	0.000	0.413	0.001	0.000	0.000
	AMR vs. EAS	0.000	0.001	0.014	0.001	0.000
	AMR vs. SAS	0.711	0.018	0.055	0.054	0.061
	EUR vs. EAS	0.000	0.002	0.217	0.640	0.645
	EUR vs. SAS	0.000	0.061	0.071	0.045	0.038
	EAS vs. SAS	0.000	0.255	0.549	0.119	0.012

Chr10	AFR vs. AMR	0.000	0.000	0.170	0.095	0.169
	AFR vs. EUR	0.000	0.000	0.000	0.000	0.000
	AFR vs. EAS	0.000	0.000	0.000	0.000	0.000
	AFR vs. SAS	0.000	0.000	0.007	0.000	0.001
	AMR vs. EUR	0.000	0.698	0.000	0.000	0.000
	AMR vs. EAS	0.000	0.208	0.000	0.000	0.000
	AMR vs. SAS	0.000	0.432	0.001	0.034	0.164
	EUR vs. EAS	0.000	0.361	0.000	0.000	0.000
	EUR vs. SAS	0.000	0.633	0.001	0.000	0.000
	EAS vs. SAS	0.013	0.648	0.000	0.000	0.000
Chr11	AFR vs. AMR	0.000	0.000	0.000	0.000	0.000
	AFR vs. EUR	0.000	0.000	0.000	0.000	0.000
	AFR vs. EAS	0.000	0.000	0.000	0.106	0.494
	AFR vs. SAS	0.000	0.000	0.014	0.111	0.633
	AMR vs. EUR	0.133	0.074	0.055	0.244	0.448
	AMR vs. EAS	0.000	0.000	0.000	0.000	0.000
	AMR vs. SAS	0.239	0.000	0.000	0.000	0.000
	EUR vs. EAS	0.000	0.000	0.000	0.000	0.000
	EUR vs. SAS	0.426	0.000	0.000	0.000	0.000
	EAS vs. SAS	0.000	0.000	0.144	0.923	0.867
Chr12	AFR vs. AMR	0.000	0.000	0.000	0.000	0.000
	AFR vs. EUR	0.000	0.000	0.000	0.000	0.000
	AFR vs. EAS	0.000	0.000	0.000	0.000	0.000
	AFR vs. SAS	0.000	0.000	0.000	0.000	0.000
	AMR vs. EUR	0.000	0.000	0.001	0.008	0.087
	AMR vs. EAS	0.950	0.000	0.000	0.000	0.000
	AMR vs. SAS	0.000	0.000	0.000	0.000	0.000
	EUR vs. EAS	0.000	0.000	0.000	0.000	0.000
	EUR vs. SAS	0.000	0.000	0.000	0.000	0.000
	EAS vs. SAS	0.000	0.000	0.000	0.000	0.000
Chr13	AFR vs. AMR	0.000	0.000	0.476	0.005	0.000
	AFR vs. EUR	0.000	0.000	0.000	0.000	0.000
	AFR vs. EAS	0.000	0.000	0.000	0.000	0.000
	AFR vs. SAS	0.000	0.000	0.000	0.000	0.000
	AMR vs. EUR	0.012	0.169	0.000	0.000	0.000
	AMR vs. EAS	0.000	0.000	0.000	0.000	0.000
	AMR vs. SAS	0.180	0.000	0.000	0.000	0.000
	EUR vs. EAS	0.000	0.000	0.000	0.000	0.000
	EUR vs. SAS	0.109	0.000	0.000	0.000	0.000
	EAS vs. SAS	0.000	0.001	0.000	0.000	0.000
Chr14	AFR vs. AMR	0.000	0.000	0.000	0.000	0.000
	AFR vs. EUR	0.000	0.000	0.000	0.000	0.000
	AFR vs. EAS	0.000	0.000	0.000	0.000	0.000
	AFR vs. SAS	0.000	0.000	0.000	0.000	0.000
	AMR vs. EUR	0.000	0.001	0.000	0.363	0.279
	AMR vs. EAS	0.000	0.000	0.007	0.000	0.000
	AMR vs. SAS	0.315	0.497	0.507	0.181	0.009

	EUR vs. EAS	0.186	0.000	0.000	0.000	0.000
	EUR vs. SAS	0.000	0.001	0.000	0.014	0.089
	EAS vs. SAS	0.000	0.000	0.000	0.008	0.034
Chr15	AFR vs. AMR	0.000	0.000	0.000	0.000	0.000
	AFR vs. EUR	0.000	0.000	0.000	0.000	0.000
	AFR vs. EAS	0.000	0.000	0.000	0.000	0.000
	AFR vs. SAS	0.000	0.000	0.000	0.000	0.000
	AMR vs. EUR	0.000	0.012	0.033	0.027	0.052
	AMR vs. EAS	0.157	0.054	0.036	0.013	0.018
	AMR vs. SAS	0.713	0.590	0.015	0.000	0.000
	EUR vs. EAS	0.000	0.000	0.000	0.000	0.000
	EUR vs. SAS	0.000	0.001	0.732	0.050	0.003
	EAS vs. SAS	0.011	0.134	0.000	0.000	0.000
Chr16	AFR vs. AMR	0.000	0.000	0.000	0.000	0.000
	AFR vs. EUR	0.000	0.000	0.000	0.000	0.000
	AFR vs. EAS	0.000	0.000	0.000	0.000	0.000
	AFR vs. SAS	0.000	0.000	0.000	0.000	0.000
	AMR vs. EUR	0.000	0.000	0.615	0.263	0.146
	AMR vs. EAS	0.000	0.000	0.000	0.000	0.000
	AMR vs. SAS	0.000	0.003	0.085	0.036	0.036
	EUR vs. EAS	0.000	0.021	0.000	0.000	0.000
	EUR vs. SAS	0.000	0.000	0.145	0.000	0.000
	EAS vs. SAS	0.000	0.000	0.000	0.000	0.000
Chr17	AFR vs. AMR	0.000	0.000	0.000	0.000	0.000
	AFR vs. EUR	0.000	0.000	0.000	0.000	0.000
	AFR vs. EAS	0.000	0.000	0.000	0.000	0.000
	AFR vs. SAS	0.000	0.000	0.000	0.000	0.000
	AMR vs. EUR	0.000	0.000	0.208	0.857	0.439
	AMR vs. EAS	0.000	0.000	0.000	0.000	0.000
	AMR vs. SAS	0.527	0.033	0.008	0.012	0.006
	EUR vs. EAS	0.000	0.000	0.000	0.000	0.000
	EUR vs. SAS	0.000	0.015	0.104	0.001	0.000
	EAS vs. SAS	0.000	0.000	0.000	0.000	0.000
Chr18	AFR vs. AMR	0.000	0.000	0.857	0.000	0.000
	AFR vs. EUR	0.000	0.000	0.374	0.000	0.000
	AFR vs. EAS	0.000	0.000	0.000	0.000	0.000
	AFR vs. SAS	0.000	0.000	0.000	0.000	0.000
	AMR vs. EUR	0.007	0.000	0.381	0.033	0.028
	AMR vs. EAS	0.000	0.001	0.000	0.000	0.000
	AMR vs. SAS	0.002	0.650	0.001	0.000	0.000
	EUR vs. EAS	0.000	0.000	0.000	0.000	0.011
	EUR vs. SAS	0.570	0.000	0.003	0.019	0.049
	EAS vs. SAS	0.000	0.000	0.009	0.247	0.635
Chr19	AFR vs. AMR	0.000	0.000	0.000	0.000	0.000
	AFR vs. EUR	0.000	0.000	0.000	0.000	0.000
	AFR vs. EAS	0.000	0.000	0.000	0.000	0.000
	AFR vs. SAS	0.000	0.000	0.000	0.000	0.000

	AMR vs. EUR	0.000	0.000	0.688	0.014	0.009
	AMR vs. EAS	0.000	0.000	0.000	0.028	0.264
	AMR vs. SAS	0.002	0.150	0.088	0.012	0.002
	EUR vs. EAS	0.000	0.000	0.000	0.000	0.000
	EUR vs. SAS	0.000	0.000	0.154	0.921	0.677
	EAS vs. SAS	0.000	0.000	0.000	0.000	0.000
Chr20	AFR vs. AMR	0.000	0.000	0.000	0.000	0.000
	AFR vs. EUR	0.000	0.000	0.000	0.000	0.000
	AFR vs. EAS	0.000	0.000	0.000	0.000	0.000
	AFR vs. SAS	0.000	0.000	0.000	0.000	0.000
	AMR vs. EUR	0.000	0.409	0.095	0.112	0.225
	AMR vs. EAS	0.000	0.008	0.000	0.000	0.000
	AMR vs. SAS	0.688	0.000	0.000	0.000	0.000
	EUR vs. EAS	0.026	0.000	0.000	0.000	0.000
	EUR vs. SAS	0.000	0.000	0.000	0.000	0.000
	EAS vs. SAS	0.000	0.000	0.000	0.007	0.106
Chr21	AFR vs. AMR	0.000	0.000	0.000	0.000	0.000
	AFR vs. EUR	0.000	0.000	0.000	0.000	0.000
	AFR vs. EAS	0.000	0.000	0.000	0.000	0.000
	AFR vs. SAS	0.000	0.000	0.000	0.000	0.000
	AMR vs. EUR	0.000	0.627	0.001	0.000	0.000
	AMR vs. EAS	0.000	0.837	0.042	0.013	0.004
	AMR vs. SAS	0.004	0.003	0.086	0.383	0.436
	EUR vs. EAS	0.000	0.330	0.000	0.000	0.000
	EUR vs. SAS	0.016	0.000	0.000	0.000	0.000
	EAS vs. SAS	0.000	0.000	0.912	0.089	0.025
Chr22	AFR vs. AMR	0.000	0.000	0.007	0.629	0.410
	AFR vs. EUR	0.000	0.000	0.000	0.000	0.000
	AFR vs. EAS	0.000	0.000	0.000	0.000	0.000
	AFR vs. SAS	0.000	0.000	0.781	0.688	0.249
	AMR vs. EUR	0.000	0.005	0.000	0.000	0.000
	AMR vs. EAS	0.000	0.981	0.000	0.000	0.000
	AMR vs. SAS	0.004	0.000	0.013	0.372	0.990
	EUR vs. EAS	0.111	0.001	0.364	0.761	0.176
	EUR vs. SAS	0.000	0.238	0.000	0.000	0.000
	EAS vs. SAS	0.000	0.000	0.000	0.000	0.000
ChrX	AFR vs. AMR	0.000	0.000	0.019	0.698	0.087
	AFR vs. EUR	0.000	0.000	0.513	0.003	0.000
	AFR vs. EAS	0.000	0.013	0.394	0.206	0.005
	AFR vs. SAS	0.000	0.000	0.532	0.013	0.000
	AMR vs. EUR	0.000	0.941	0.059	0.039	0.040
	AMR vs. EAS	0.163	0.050	0.043	0.079	0.090
	AMR vs. SAS	0.952	0.139	0.183	0.289	0.279
	EUR vs. EAS	0.000	0.268	0.582	0.386	0.371
	EUR vs. SAS	0.000	0.029	0.606	0.302	0.336
	EAS vs. SAS	0.051	0.347	0.197	0.204	0.239
ChrY	AFR vs. AMR	0.000	0.000	0.000	0.002	0.008

	AFR vs. EUR	0.000	0.000	0.000	0.000	0.000
	AFR vs. EAS	0.000	0.000	0.000	0.000	0.000
	AFR vs. SAS	0.000	0.000	0.000	0.000	0.000
	AMR vs. EUR	0.010	0.012	0.163	0.094	0.045
	AMR vs. EAS	0.000	0.000	0.000	0.000	0.000
	AMR vs. SAS	0.000	0.000	0.000	0.000	0.000
	EUR vs. EAS	0.000	0.000	0.000	0.000	0.000
	EUR vs. SAS	0.000	0.000	0.000	0.000	0.000
	EAS vs. SAS	0.000	0.000	0.000	0.022	0.004
Percentage (%) of Pairs with Significant Differences	AFR vs. AMR	100.0	100.0	87.5	83.3	79.2
	AFR vs. EUR	100.0	100.0	91.7	95.8	87.5
	AFR vs. EAS	100.0	100.0	95.8	91.7	91.7
	AFR vs. SAS	100.0	100.0	91.7	83.3	83.3
	AMR vs. EUR	95.8	66.7	58.3	70.8	62.5
	AMR vs. EAS	87.5	75.0	91.7	91.7	83.3
	AMR vs. SAS	62.5	58.3	66.7	66.7	66.7
	EUR vs. EAS	87.5	79.2	70.8	83.3	87.5
	EUR vs. SAS	87.5	79.2	62.5	75.0	79.2
	EAS vs. SAS	91.7	70.8	79.2	75.0	83.3

Table S4. SNP-alpha diversity of each individual in the 1000-Genomes Project (Excel file)

Table S5. The *p*-value of Wilcoxon tests for the SNP-alpha diversity of whole genome among different populations

**Treatments**	***q*=0**	***q*=1**	***q*=2**	***q*=3**	***q*=4**
AFR vs. AMR	0.000	0.000	0.000	0.000	0.000
AFR vs. EUR	0.000	0.000	0.000	0.000	0.000
AFR vs. EAS	0.000	0.000	0.000	0.000	0.000
AFR vs. SAS	0.000	0.000	0.000	0.000	0.000
AMR vs. EUR	0.000	0.000	0.155	0.161	0.911
AMR vs. EAS	0.000	0.000	0.000	0.000	0.000
AMR vs. SAS	0.026	0.883	0.512	0.820	0.871
EUR vs. EAS	0.368	0.176	0.000	0.000	0.000
EUR vs. SAS	0.000	0.000	0.529	0.123	0.895
EAS vs. SAS	0.000	0.000	0.000	0.000	0.000
**% of Pairs with Significant Differences**	**90**	**80**	**70**	**70**	**70**

Table S6. The means of pair-wise, chromosome-level SNP beta-diversity and similarity measures between any two individuals belonging to two different populations [chromosome level=compared SNPs on the two corresponding (*i.e.*, the same numbered) chromosomes from two individuals belonging to different populations]

Treats	Chromosome	*q*=0					*q*=1					*q*=2				
		Beta	Four Similarity Measures				*Beta*	Four Similarity Measures				*Beta*	Four Similarity Measures			
			C_q	U_q	S_q	V_q		C_q	U_q	S_q	V_q		C_q	U_q	S_q	V_q
AFR vs. AMR	Chr1	1.054	0.946	0.897	0.897	0.946	1.036	0.948	0.948	0.930	0.964	1.028	0.945	0.972	0.945	0.972
	Chr2	1.055	0.945	0.896	0.896	0.945	1.036	0.949	0.949	0.930	0.964	1.030	0.942	0.970	0.942	0.970
	Chr3	1.049	0.951	0.907	0.907	0.951	1.034	0.951	0.951	0.933	0.966	1.028	0.945	0.972	0.945	0.972
	Chr4	1.043	0.957	0.917	0.917	0.957	1.033	0.954	0.954	0.937	0.967	1.029	0.943	0.971	0.943	0.971
	Chr5	1.052	0.948	0.901	0.901	0.948	1.036	0.949	0.949	0.930	0.964	1.032	0.937	0.968	0.937	0.968
	Chr6	1.051	0.949	0.903	0.903	0.949	1.037	0.948	0.948	0.929	0.963	1.033	0.936	0.967	0.936	0.967
	Chr7	1.059	0.941	0.889	0.889	0.941	1.036	0.949	0.949	0.930	0.964	1.028	0.946	0.972	0.946	0.972
	Chr8	1.052	0.948	0.901	0.901	0.948	1.037	0.948	0.948	0.929	0.963	1.021	0.958	0.979	0.958	0.979
	Chr9	1.058	0.942	0.891	0.891	0.942	1.035	0.950	0.950	0.932	0.965	1.025	0.952	0.975	0.952	0.975
	Chr10	1.052	0.948	0.901	0.901	0.948	1.035	0.950	0.950	0.932	0.965	1.027	0.948	0.973	0.948	0.973
	Chr11	1.051	0.949	0.903	0.903	0.949	1.033	0.953	0.953	0.936	0.967	1.026	0.949	0.974	0.949	0.974
	Chr12	1.048	0.952	0.909	0.909	0.952	1.035	0.950	0.950	0.932	0.965	1.032	0.937	0.968	0.937	0.968
	Chr13	1.042	0.958	0.919	0.919	0.958	1.025	0.964	0.964	0.951	0.975	1.022	0.957	0.978	0.957	0.978
	Chr14	1.062	0.938	0.884	0.884	0.938	1.036	0.949	0.949	0.930	0.964	1.028	0.945	0.972	0.945	0.972
	Chr15	1.055	0.945	0.895	0.895	0.945	1.034	0.952	0.952	0.935	0.966	1.029	0.943	0.971	0.943	0.971
	Chr16	1.063	0.937	0.881	0.881	0.937	1.035	0.951	0.951	0.933	0.965	1.015	0.970	0.985	0.970	0.985
	Chr17	1.062	0.938	0.883	0.883	0.938	1.043	0.939	0.939	0.917	0.957	1.036	0.931	0.964	0.931	0.964
	Chr18	1.042	0.958	0.919	0.919	0.958	1.027	0.961	0.961	0.947	0.973	1.026	0.948	0.974	0.948	0.974
	Chr19	1.062	0.938	0.884	0.884	0.938	1.060	0.916	0.916	0.887	0.940	1.052	0.901	0.948	0.901	0.948
	Chr20	1.052	0.948	0.900	0.900	0.948	1.042	0.941	0.941	0.920	0.958	1.027	0.948	0.973	0.948	0.973
	Chr21	1.047	0.953	0.911	0.911	0.953	1.031	0.957	0.957	0.941	0.969	1.031	0.941	0.969	0.941	0.969
	Chr22	1.062	0.938	0.883	0.883	0.938	1.041	0.942	0.942	0.921	0.959	1.031	0.940	0.969	0.940	0.969
	ChrX	1.497	0.503	0.463	0.463	0.503	1.452	0.523	0.523	0.498	0.548	1.445	0.500	0.555	0.500	0.555
	ChrY	1.403	0.597	0.477	0.477	0.597	1.337	0.611	0.611	0.559	0.663	1.347	0.558	0.653	0.558	0.653
AFR vs. EUR	Chr1	1.057	0.943	0.892	0.892	0.943	1.039	0.944	0.944	0.924	0.961	1.031	0.940	0.969	0.940	0.969
	Chr2	1.057	0.943	0.892	0.892	0.943	1.039	0.945	0.945	0.925	0.961	1.033	0.936	0.967	0.936	0.967
	Chr3	1.050	0.950	0.904	0.904	0.950	1.038	0.946	0.946	0.927	0.962	1.031	0.939	0.969	0.939	0.969
	Chr4	1.045	0.955	0.914	0.914	0.955	1.036	0.949	0.949	0.931	0.964	1.033	0.936	0.967	0.936	0.967
	Chr5	1.054	0.946	0.898	0.898	0.946	1.038	0.946	0.946	0.926	0.962	1.035	0.932	0.965	0.932	0.965
	Chr6	1.056	0.944	0.893	0.893	0.944	1.041	0.943	0.943	0.922	0.959	1.036	0.931	0.964	0.931	0.964
	Chr7	1.061	0.939	0.886	0.886	0.939	1.039	0.945	0.945	0.925	0.961	1.031	0.939	0.969	0.939	0.969
	Chr8	1.054	0.946	0.898	0.898	0.946	1.039	0.945	0.945	0.925	0.961	1.022	0.956	0.978	0.956	0.978
	Chr9	1.059	0.941	0.888	0.888	0.941	1.038	0.946	0.946	0.927	0.962	1.029	0.944	0.971	0.944	0.971
	Chr10	1.055	0.945	0.896	0.896	0.945	1.037	0.947	0.947	0.928	0.963	1.030	0.943	0.970	0.943	0.970
	Chr11	1.052	0.948	0.902	0.902	0.948	1.034	0.952	0.952	0.935	0.966	1.026	0.949	0.974	0.949	0.974
	Chr12	1.049	0.951	0.906	0.906	0.951	1.037	0.948	0.948	0.929	0.963	1.034	0.934	0.966	0.934	0.966
	Chr13	1.044	0.956	0.916	0.916	0.956	1.027	0.962	0.962	0.948	0.973	1.024	0.953	0.976	0.953	0.976
	Chr14	1.064	0.936	0.879	0.879	0.936	1.040	0.943	0.943	0.923	0.960	1.035	0.933	0.965	0.933	0.965
	Chr15	1.057	0.943	0.892	0.892	0.943	1.036	0.950	0.950	0.931	0.964	1.030	0.941	0.970	0.941	0.970
	Chr16	1.065	0.935	0.878	0.878	0.935	1.037	0.947	0.947	0.928	0.963	1.017	0.967	0.983	0.967	0.983
	Chr17	1.065	0.935	0.879	0.879	0.935	1.047	0.934	0.934	0.910	0.953	1.039	0.925	0.961	0.925	0.961
	Chr18	1.044	0.956	0.916	0.916	0.956	1.029	0.958	0.958	0.943	0.971	1.030	0.943	0.970	0.943	0.970
	Chr19	1.064	0.936	0.879	0.879	0.936	1.064	0.911	0.911	0.880	0.936	1.056	0.894	0.944	0.894	0.944
	Chr20	1.055	0.945	0.896	0.896	0.945	1.045	0.936	0.936	0.914	0.955	1.030	0.941	0.970	0.941	0.970
	Chr21	1.047	0.953	0.910	0.910	0.953	1.032	0.955	0.955	0.938	0.968	1.031	0.939	0.969	0.939	0.969
	Chr22	1.066	0.934	0.877	0.877	0.934	1.045	0.937	0.937	0.915	0.955	1.037	0.928	0.963	0.928	0.963
	ChrX	1.500	0.500	0.457	0.457	0.500	1.454	0.519	0.519	0.493	0.546	1.445	0.494	0.555	0.494	0.555
	ChrY	1.436	0.564	0.442	0.442	0.564	1.365	0.582	0.582	0.529	0.635	1.374	0.528	0.626	0.528	0.626
AFR vs. EAS	Chr1	1.055	0.945	0.896	0.896	0.945	1.037	0.947	0.947	0.928	0.963	1.030	0.942	0.970	0.942	0.970
	Chr2	1.057	0.943	0.892	0.892	0.943	1.039	0.945	0.945	0.926	0.961	1.032	0.938	0.968	0.938	0.968
	Chr3	1.051	0.949	0.904	0.904	0.949	1.037	0.948	0.948	0.929	0.963	1.031	0.940	0.969	0.940	0.969

	Chr4	1.044	0.956	0.915	0.915	0.956	1.034	0.952	0.952	0.934	0.966	1.030	0.941	0.970	0.941	0.970
	Chr5	1.056	0.944	0.893	0.893	0.944	1.043	0.940	0.940	0.918	0.957	1.038	0.926	0.962	0.926	0.962
	Chr6	1.052	0.948	0.901	0.901	0.948	1.038	0.946	0.946	0.927	0.962	1.033	0.935	0.967	0.935	0.967
	Chr7	1.062	0.938	0.884	0.884	0.938	1.038	0.947	0.947	0.928	0.962	1.031	0.940	0.969	0.940	0.969
	Chr8	1.054	0.946	0.897	0.897	0.946	1.040	0.944	0.944	0.924	0.960	1.024	0.953	0.976	0.953	0.976
	Chr9	1.058	0.942	0.890	0.890	0.942	1.036	0.948	0.948	0.930	0.964	1.027	0.947	0.973	0.947	0.973
	Chr10	1.053	0.947	0.899	0.899	0.947	1.038	0.947	0.947	0.928	0.962	1.029	0.943	0.971	0.943	0.971
	Chr11	1.053	0.947	0.899	0.899	0.947	1.035	0.950	0.950	0.932	0.965	1.027	0.947	0.973	0.947	0.973
	Chr12	1.048	0.952	0.909	0.909	0.952	1.036	0.949	0.949	0.931	0.964	1.035	0.933	0.965	0.933	0.965
	Chr13	1.044	0.956	0.915	0.915	0.956	1.027	0.962	0.962	0.948	0.973	1.023	0.955	0.977	0.955	0.977
	Chr14	1.064	0.936	0.880	0.880	0.936	1.037	0.948	0.948	0.929	0.963	1.028	0.946	0.972	0.946	0.972
	Chr15	1.056	0.944	0.894	0.894	0.944	1.035	0.950	0.950	0.932	0.965	1.031	0.940	0.969	0.940	0.969
	Chr16	1.066	0.934	0.876	0.876	0.934	1.036	0.948	0.948	0.930	0.964	1.016	0.969	0.984	0.969	0.984
	Chr17	1.067	0.933	0.875	0.875	0.933	1.047	0.933	0.933	0.910	0.953	1.038	0.926	0.962	0.926	0.962
	Chr18	1.044	0.956	0.915	0.915	0.956	1.028	0.961	0.961	0.946	0.972	1.027	0.947	0.973	0.947	0.973
	Chr19	1.066	0.934	0.877	0.877	0.934	1.066	0.908	0.908	0.876	0.934	1.057	0.892	0.943	0.892	0.943
	Chr20	1.055	0.945	0.895	0.895	0.945	1.042	0.940	0.940	0.919	0.958	1.028	0.946	0.972	0.946	0.972
	Chr21	1.050	0.950	0.906	0.906	0.950	1.033	0.954	0.954	0.937	0.967	1.034	0.935	0.966	0.935	0.966
	Chr22	1.067	0.933	0.875	0.875	0.933	1.044	0.937	0.937	0.915	0.956	1.036	0.931	0.964	0.931	0.964
	ChrX	1.497	0.503	0.463	0.463	0.503	1.453	0.522	0.522	0.498	0.547	1.450	0.495	0.550	0.495	0.550
	ChrY	1.176	0.824	0.711	0.711	0.824	1.100	0.868	0.868	0.831	0.900	1.092	0.844	0.908	0.844	0.908
AFR vs. SAS	Chr1	1.055	0.945	0.896	0.896	0.945	1.037	0.947	0.947	0.928	0.963	1.029	0.944	0.971	0.944	0.971
	Chr2	1.056	0.944	0.895	0.895	0.944	1.037	0.948	0.948	0.930	0.963	1.030	0.941	0.970	0.941	0.970
	Chr3	1.050	0.950	0.905	0.905	0.950	1.036	0.950	0.950	0.931	0.964	1.030	0.943	0.970	0.943	0.970
	Chr4	1.043	0.957	0.917	0.917	0.957	1.033	0.953	0.953	0.936	0.967	1.029	0.943	0.971	0.943	0.971
	Chr5	1.052	0.948	0.900	0.900	0.948	1.036	0.948	0.948	0.930	0.964	1.033	0.936	0.967	0.936	0.967
	Chr6	1.052	0.948	0.900	0.900	0.948	1.037	0.947	0.947	0.928	0.963	1.033	0.935	0.967	0.935	0.967
	Chr7	1.059	0.941	0.888	0.888	0.941	1.036	0.949	0.949	0.931	0.964	1.027	0.947	0.973	0.947	0.973
	Chr8	1.051	0.949	0.902	0.902	0.949	1.036	0.949	0.949	0.930	0.964	1.021	0.959	0.979	0.959	0.979
	Chr9	1.057	0.943	0.893	0.893	0.943	1.035	0.951	0.951	0.933	0.965	1.025	0.951	0.975	0.951	0.975
	Chr10	1.054	0.946	0.898	0.898	0.946	1.035	0.950	0.950	0.932	0.965	1.027	0.947	0.973	0.947	0.973
	Chr11	1.051	0.949	0.903	0.903	0.949	1.032	0.954	0.954	0.937	0.968	1.024	0.952	0.976	0.952	0.976
	Chr12	1.046	0.954	0.911	0.911	0.954	1.035	0.951	0.951	0.933	0.965	1.033	0.937	0.967	0.937	0.967
	Chr13	1.043	0.957	0.917	0.917	0.957	1.026	0.964	0.964	0.950	0.974	1.021	0.958	0.979	0.958	0.979
	Chr14	1.062	0.938	0.883	0.883	0.938	1.036	0.950	0.950	0.931	0.964	1.027	0.947	0.973	0.947	0.973
	Chr15	1.056	0.944	0.893	0.893	0.944	1.034	0.951	0.951	0.934	0.966	1.029	0.943	0.971	0.943	0.971
	Chr16	1.061	0.939	0.885	0.885	0.939	1.034	0.952	0.952	0.935	0.966	1.015	0.971	0.985	0.971	0.985
	Chr17	1.062	0.938	0.883	0.883	0.938	1.044	0.938	0.938	0.916	0.956	1.036	0.930	0.964	0.930	0.964
	Chr18	1.043	0.957	0.917	0.917	0.957	1.027	0.961	0.961	0.947	0.973	1.027	0.948	0.973	0.948	0.973
	Chr19	1.061	0.939	0.886	0.886	0.939	1.059	0.918	0.918	0.889	0.941	1.052	0.902	0.948	0.902	0.948
	Chr20	1.052	0.948	0.901	0.901	0.948	1.041	0.943	0.943	0.922	0.959	1.028	0.946	0.972	0.946	0.972
	Chr21	1.047	0.953	0.910	0.910	0.953	1.030	0.958	0.958	0.943	0.970	1.029	0.943	0.971	0.943	0.971
	Chr22	1.062	0.938	0.883	0.883	0.938	1.041	0.943	0.943	0.922	0.959	1.030	0.941	0.970	0.941	0.970
	ChrX	1.495	0.505	0.466	0.466	0.505	1.451	0.524	0.524	0.501	0.549	1.446	0.500	0.554	0.500	0.554
	ChrY	1.219	0.781	0.653	0.653	0.781	1.138	0.819	0.819	0.770	0.862	1.145	0.764	0.855	0.764	0.855
AMR vs. EUR	Chr1	1.051	0.949	0.902	0.902	0.949	1.033	0.953	0.953	0.936	0.967	1.024	0.953	0.976	0.953	0.976
	Chr2	1.053	0.947	0.898	0.898	0.947	1.034	0.952	0.952	0.934	0.966	1.025	0.951	0.975	0.951	0.975
	Chr3	1.047	0.953	0.910	0.910	0.953	1.033	0.953	0.953	0.936	0.967	1.024	0.953	0.976	0.953	0.976
	Chr4	1.040	0.960	0.923	0.923	0.960	1.030	0.957	0.957	0.942	0.970	1.024	0.952	0.976	0.952	0.976
	Chr5	1.051	0.949	0.903	0.903	0.949	1.034	0.952	0.952	0.934	0.966	1.027	0.947	0.973	0.947	0.973
	Chr6	1.055	0.945	0.897	0.897	0.945	1.037	0.948	0.948	0.929	0.963	1.029	0.943	0.971	0.943	0.971
	Chr7	1.059	0.941	0.889	0.889	0.941	1.034	0.952	0.952	0.934	0.966	1.022	0.956	0.978	0.956	0.978
	Chr8	1.050	0.950	0.905	0.905	0.950	1.033	0.953	0.953	0.936	0.967	1.016	0.969	0.984	0.969	0.984
	Chr9	1.055	0.945	0.896	0.896	0.945	1.032	0.954	0.954	0.938	0.968	1.020	0.961	0.980	0.961	0.980
	Chr10	1.051	0.949	0.903	0.903	0.949	1.033	0.953	0.953	0.936	0.967	1.023	0.955	0.977	0.955	0.977
	Chr11	1.047	0.953	0.910	0.910	0.953	1.029	0.959	0.959	0.943	0.971	1.020	0.961	0.980	0.961	0.980
	Chr12	1.045	0.955	0.913	0.913	0.955	1.032	0.955	0.955	0.938	0.968	1.027	0.948	0.973	0.948	0.973
	Chr13	1.038	0.962	0.927	0.927	0.962	1.022	0.969	0.969	0.957	0.978	1.019	0.963	0.981	0.963	0.981
	Chr14	1.057	0.943	0.892	0.892	0.943	1.034	0.952	0.952	0.935	0.966	1.027	0.948	0.973	0.948	0.973

	Chr15	1.052	0.948	0.902	0.902	0.948	1.030	0.957	0.957	0.942	0.970	1.023	0.955	0.977	0.955	0.977
	Chr16	1.062	0.938	0.884	0.884	0.938	1.032	0.955	0.955	0.939	0.968	1.011	0.979	0.989	0.979	0.989
	Chr17	1.061	0.939	0.886	0.886	0.939	1.042	0.941	0.941	0.920	0.958	1.030	0.942	0.970	0.942	0.970
	Chr18	1.038	0.962	0.926	0.926	0.962	1.025	0.965	0.965	0.952	0.975	1.022	0.958	0.978	0.958	0.978
	Chr19	1.065	0.935	0.878	0.878	0.935	1.059	0.917	0.917	0.889	0.941	1.047	0.910	0.953	0.910	0.953
	Chr20	1.054	0.946	0.898	0.898	0.946	1.040	0.944	0.944	0.924	0.960	1.021	0.959	0.979	0.959	0.979
	Chr21	1.045	0.955	0.914	0.914	0.955	1.027	0.961	0.961	0.947	0.973	1.023	0.956	0.977	0.956	0.977
	Chr22	1.065	0.935	0.878	0.878	0.935	1.041	0.943	0.943	0.922	0.959	1.026	0.949	0.974	0.949	0.974
	ChrX	1.491	0.509	0.467	0.467	0.509	1.440	0.535	0.535	0.511	0.560	1.430	0.520	0.570	0.520	0.570
	ChrY	1.474	0.526	0.386	0.386	0.526	1.345	0.594	0.594	0.531	0.655	1.298	0.605	0.702	0.605	0.702
	Chr1	1.050	0.950	0.904	0.904	0.950	1.032	0.954	0.954	0.937	0.968	1.024	0.954	0.976	0.954	0.976
	Chr2	1.053	0.947	0.899	0.899	0.947	1.034	0.952	0.952	0.935	0.966	1.025	0.952	0.975	0.952	0.975
	Chr3	1.047	0.953	0.910	0.910	0.953	1.033	0.954	0.954	0.937	0.967	1.024	0.954	0.976	0.954	0.976
	Chr4	1.041	0.959	0.922	0.922	0.959	1.030	0.958	0.958	0.942	0.970	1.024	0.953	0.976	0.953	0.976
	Chr5	1.054	0.946	0.897	0.897	0.946	1.037	0.947	0.947	0.928	0.963	1.029	0.943	0.971	0.943	0.971
	Chr6	1.050	0.950	0.904	0.904	0.950	1.035	0.950	0.950	0.932	0.965	1.029	0.944	0.971	0.944	0.971
	Chr7	1.059	0.941	0.889	0.889	0.941	1.033	0.953	0.953	0.935	0.967	1.022	0.956	0.978	0.956	0.978
	Chr8	1.049	0.951	0.907	0.907	0.951	1.033	0.953	0.953	0.936	0.967	1.016	0.969	0.984	0.969	0.984
	Chr9	1.056	0.944	0.895	0.895	0.944	1.033	0.954	0.954	0.937	0.967	1.020	0.961	0.980	0.961	0.980
	Chr10	1.051	0.949	0.903	0.903	0.949	1.034	0.952	0.952	0.935	0.966	1.023	0.954	0.977	0.954	0.977
	Chr11	1.048	0.952	0.908	0.908	0.952	1.030	0.957	0.957	0.942	0.970	1.021	0.960	0.979	0.960	0.979
AMR vs. EAS	Chr12	1.045	0.955	0.914	0.914	0.955	1.032	0.954	0.954	0.937	0.968	1.028	0.945	0.972	0.945	0.972
	Chr13	1.039	0.961	0.925	0.925	0.961	1.022	0.968	0.968	0.956	0.978	1.018	0.964	0.982	0.964	0.982
	Chr14	1.059	0.941	0.888	0.888	0.941	1.035	0.951	0.951	0.933	0.965	1.026	0.950	0.974	0.950	0.974
	Chr15	1.052	0.948	0.901	0.901	0.948	1.030	0.957	0.957	0.941	0.970	1.023	0.955	0.977	0.955	0.977
	Chr16	1.062	0.938	0.883	0.883	0.938	1.031	0.955	0.955	0.939	0.969	1.010	0.980	0.990	0.980	0.990
	Chr17	1.063	0.937	0.881	0.881	0.937	1.042	0.941	0.941	0.919	0.958	1.030	0.942	0.970	0.942	0.970
	Chr18	1.039	0.961	0.925	0.925	0.961	1.024	0.966	0.966	0.953	0.976	1.021	0.959	0.979	0.959	0.979
	Chr19	1.068	0.932	0.873	0.873	0.932	1.062	0.913	0.913	0.883	0.938	1.049	0.906	0.951	0.906	0.951
	Chr20	1.054	0.946	0.897	0.897	0.946	1.038	0.946	0.946	0.927	0.962	1.020	0.961	0.980	0.961	0.980
	Chr21	1.048	0.952	0.909	0.909	0.952	1.028	0.960	0.960	0.945	0.972	1.024	0.954	0.976	0.954	0.976
	Chr22	1.067	0.933	0.875	0.875	0.933	1.040	0.943	0.943	0.923	0.960	1.025	0.951	0.975	0.951	0.975
	ChrX	1.499	0.501	0.462	0.462	0.501	1.448	0.528	0.528	0.505	0.552	1.441	0.510	0.559	0.510	0.559
	ChrY	1.392	0.608	0.480	0.480	0.608	1.308	0.637	0.637	0.582	0.692	1.289	0.612	0.711	0.612	0.711
	Chr1	1.051	0.949	0.903	0.903	0.949	1.033	0.953	0.953	0.936	0.967	1.024	0.953	0.976	0.953	0.976
	Chr2	1.053	0.947	0.899	0.899	0.947	1.033	0.953	0.953	0.935	0.967	1.024	0.952	0.976	0.952	0.976
	Chr3	1.047	0.953	0.910	0.910	0.953	1.032	0.954	0.954	0.937	0.968	1.023	0.954	0.977	0.954	0.977
	Chr4	1.040	0.960	0.923	0.923	0.960	1.030	0.958	0.958	0.942	0.970	1.024	0.953	0.976	0.953	0.976
	Chr5	1.051	0.949	0.902	0.902	0.949	1.034	0.952	0.952	0.934	0.966	1.027	0.947	0.973	0.947	0.973
	Chr6	1.052	0.948	0.901	0.901	0.948	1.036	0.949	0.949	0.931	0.964	1.029	0.944	0.971	0.944	0.971
	Chr7	1.058	0.942	0.890	0.890	0.942	1.033	0.953	0.953	0.935	0.967	1.021	0.958	0.979	0.958	0.979
	Chr8	1.049	0.951	0.906	0.906	0.951	1.033	0.954	0.954	0.937	0.967	1.016	0.969	0.984	0.969	0.984
	Chr9	1.055	0.945	0.896	0.896	0.945	1.032	0.955	0.955	0.938	0.968	1.020	0.961	0.980	0.961	0.980
	Chr10	1.051	0.949	0.902	0.902	0.949	1.033	0.953	0.953	0.936	0.967	1.022	0.956	0.978	0.956	0.978
	Chr11	1.047	0.953	0.910	0.910	0.953	1.029	0.959	0.959	0.943	0.971	1.020	0.961	0.980	0.961	0.980
AMR vs. SAS	Chr12	1.045	0.955	0.915	0.915	0.955	1.032	0.955	0.955	0.939	0.968	1.027	0.947	0.973	0.947	0.973
	Chr13	1.039	0.961	0.926	0.926	0.961	1.022	0.969	0.969	0.957	0.978	1.018	0.964	0.982	0.964	0.982
	Chr14	1.057	0.943	0.891	0.891	0.943	1.033	0.953	0.953	0.936	0.967	1.024	0.952	0.976	0.952	0.976
	Chr15	1.053	0.947	0.900	0.900	0.947	1.030	0.957	0.957	0.941	0.970	1.024	0.954	0.976	0.954	0.976
	Chr16	1.061	0.939	0.886	0.886	0.939	1.031	0.956	0.956	0.941	0.969	1.010	0.980	0.990	0.980	0.990
	Chr17	1.061	0.939	0.886	0.886	0.939	1.040	0.943	0.943	0.923	0.960	1.029	0.943	0.971	0.943	0.971
	Chr18	1.039	0.961	0.925	0.925	0.961	1.024	0.965	0.965	0.952	0.976	1.021	0.959	0.979	0.959	0.979
	Chr19	1.064	0.936	0.880	0.880	0.936	1.058	0.918	0.918	0.890	0.942	1.048	0.909	0.952	0.909	0.952
	Chr20	1.053	0.947	0.900	0.900	0.947	1.038	0.946	0.946	0.927	0.962	1.020	0.960	0.980	0.960	0.980
	Chr21	1.045	0.955	0.913	0.913	0.955	1.027	0.962	0.962	0.947	0.973	1.022	0.956	0.978	0.956	0.978
	Chr22	1.064	0.936	0.880	0.880	0.936	1.039	0.944	0.944	0.924	0.961	1.024	0.953	0.976	0.953	0.976
	ChrX	1.500	0.500	0.461	0.461	0.500	1.449	0.527	0.527	0.504	0.551	1.440	0.512	0.560	0.512	0.560
	ChrY	1.395	0.605	0.470	0.470	0.605	1.297	0.645	0.645	0.585	0.703	1.264	0.630	0.736	0.630	0.736
EUR vs. EAS	Chr1	1.053	0.947	0.899	0.899	0.947	1.035	0.951	0.951	0.933	0.965	1.025	0.952	0.975	0.952	0.975

	Chr2	1.056	0.944	0.893	0.893	0.944	1.036	0.948	0.948	0.930	0.964	1.026	0.949	0.974	0.949	0.974
	Chr3	1.049	0.951	0.906	0.906	0.951	1.034	0.951	0.951	0.933	0.966	1.024	0.953	0.976	0.953	0.976
	Chr4	1.042	0.958	0.920	0.920	0.958	1.031	0.956	0.956	0.940	0.969	1.025	0.952	0.975	0.952	0.975
	Chr5	1.056	0.944	0.894	0.894	0.944	1.039	0.945	0.945	0.925	0.961	1.030	0.942	0.970	0.942	0.970
	Chr6	1.055	0.945	0.895	0.895	0.945	1.038	0.946	0.946	0.927	0.962	1.031	0.941	0.969	0.941	0.969
	Chr7	1.061	0.939	0.885	0.885	0.939	1.035	0.950	0.950	0.932	0.965	1.023	0.956	0.977	0.956	0.977
	Chr8	1.051	0.949	0.903	0.903	0.949	1.035	0.951	0.951	0.933	0.965	1.016	0.969	0.984	0.969	0.984
	Chr9	1.058	0.942	0.891	0.891	0.942	1.034	0.951	0.951	0.933	0.966	1.021	0.960	0.979	0.960	0.979
	Chr10	1.054	0.946	0.898	0.898	0.946	1.035	0.950	0.950	0.931	0.965	1.024	0.952	0.976	0.952	0.976
	Chr11	1.050	0.950	0.905	0.905	0.950	1.031	0.956	0.956	0.940	0.969	1.020	0.960	0.980	0.960	0.980
	Chr12	1.047	0.953	0.910	0.910	0.953	1.034	0.951	0.951	0.934	0.966	1.029	0.943	0.971	0.943	0.971
	Chr13	1.041	0.959	0.922	0.922	0.959	1.023	0.967	0.967	0.954	0.977	1.019	0.962	0.981	0.962	0.981
	Chr14	1.061	0.939	0.885	0.885	0.939	1.037	0.948	0.948	0.929	0.963	1.028	0.946	0.972	0.946	0.972
	Chr15	1.055	0.945	0.896	0.896	0.945	1.033	0.954	0.954	0.937	0.967	1.024	0.953	0.976	0.953	0.976
	Chr16	1.064	0.936	0.880	0.880	0.936	1.033	0.954	0.954	0.937	0.967	1.010	0.980	0.990	0.980	0.990
	Chr17	1.066	0.934	0.876	0.876	0.934	1.044	0.938	0.938	0.916	0.956	1.030	0.941	0.970	0.941	0.970
	Chr18	1.041	0.959	0.922	0.922	0.959	1.025	0.964	0.964	0.951	0.975	1.021	0.958	0.979	0.958	0.979
	Chr19	1.071	0.929	0.868	0.868	0.929	1.064	0.910	0.910	0.879	0.936	1.051	0.904	0.949	0.904	0.949
	Chr20	1.056	0.944	0.893	0.893	0.944	1.041	0.943	0.943	0.922	0.959	1.021	0.960	0.979	0.960	0.979
	Chr21	1.049	0.951	0.907	0.907	0.951	1.029	0.958	0.958	0.943	0.971	1.024	0.953	0.976	0.953	0.976
	Chr22	1.070	0.930	0.870	0.870	0.930	1.042	0.940	0.940	0.919	0.958	1.027	0.948	0.973	0.948	0.973
	ChrX	1.502	0.498	0.457	0.457	0.498	1.449	0.526	0.526	0.502	0.551	1.439	0.511	0.561	0.511	0.561
	ChrY	1.411	0.589	0.467	0.467	0.589	1.329	0.618	0.618	0.565	0.671	1.306	0.599	0.694	0.599	0.694
EUR vs. SAS	Chr1	1.052	0.948	0.902	0.902	0.948	1.033	0.953	0.953	0.936	0.967	1.024	0.954	0.976	0.954	0.976
	Chr2	1.054	0.946	0.898	0.898	0.946	1.034	0.952	0.952	0.935	0.966	1.024	0.953	0.976	0.953	0.976
	Chr3	1.047	0.953	0.910	0.910	0.953	1.033	0.953	0.953	0.936	0.967	1.023	0.955	0.977	0.955	0.977
	Chr4	1.040	0.960	0.923	0.923	0.960	1.030	0.958	0.958	0.942	0.970	1.024	0.953	0.976	0.953	0.976
	Chr5	1.051	0.949	0.903	0.903	0.949	1.034	0.952	0.952	0.935	0.966	1.027	0.948	0.973	0.948	0.973
	Chr6	1.055	0.945	0.896	0.896	0.945	1.036	0.949	0.949	0.930	0.964	1.029	0.944	0.971	0.944	0.971
	Chr7	1.059	0.941	0.889	0.889	0.941	1.033	0.953	0.953	0.935	0.967	1.021	0.960	0.979	0.960	0.979
	Chr8	1.049	0.951	0.906	0.906	0.951	1.033	0.954	0.954	0.937	0.967	1.015	0.970	0.985	0.970	0.985
	Chr9	1.055	0.945	0.896	0.896	0.945	1.032	0.954	0.954	0.937	0.968	1.020	0.961	0.980	0.961	0.980
	Chr10	1.052	0.948	0.901	0.901	0.948	1.033	0.953	0.953	0.936	0.967	1.022	0.956	0.978	0.956	0.978
	Chr11	1.047	0.953	0.911	0.911	0.953	1.028	0.960	0.960	0.945	0.972	1.019	0.963	0.981	0.963	0.981
	Chr12	1.045	0.955	0.914	0.914	0.955	1.032	0.955	0.955	0.938	0.968	1.027	0.948	0.973	0.948	0.973
	Chr13	1.039	0.961	0.926	0.926	0.961	1.022	0.969	0.969	0.957	0.978	1.019	0.964	0.981	0.964	0.981
	Chr14	1.057	0.943	0.893	0.893	0.943	1.033	0.953	0.953	0.936	0.967	1.025	0.951	0.975	0.951	0.975
	Chr15	1.053	0.947	0.900	0.900	0.947	1.030	0.957	0.957	0.941	0.970	1.023	0.956	0.977	0.956	0.977
	Chr16	1.060	0.940	0.887	0.887	0.940	1.030	0.957	0.957	0.941	0.970	1.010	0.981	0.990	0.981	0.990
	Chr17	1.061	0.939	0.885	0.885	0.939	1.040	0.943	0.943	0.922	0.960	1.029	0.944	0.971	0.944	0.971
	Chr18	1.039	0.961	0.925	0.925	0.961	1.024	0.965	0.965	0.953	0.976	1.020	0.960	0.980	0.960	0.980
	Chr19	1.064	0.936	0.879	0.879	0.936	1.058	0.918	0.918	0.890	0.942	1.048	0.909	0.952	0.909	0.952
	Chr20	1.053	0.947	0.899	0.899	0.947	1.039	0.945	0.945	0.926	0.961	1.020	0.961	0.980	0.961	0.980
	Chr21	1.044	0.956	0.915	0.915	0.956	1.026	0.962	0.962	0.948	0.974	1.021	0.958	0.979	0.958	0.979
	Chr22	1.065	0.935	0.877	0.877	0.935	1.041	0.942	0.942	0.921	0.959	1.027	0.948	0.973	0.948	0.973
	ChrX	1.501	0.499	0.458	0.458	0.499	1.448	0.527	0.527	0.504	0.552	1.436	0.514	0.564	0.514	0.564
	ChrY	1.415	0.585	0.455	0.455	0.585	1.316	0.625	0.625	0.566	0.684	1.276	0.618	0.724	0.618	0.724
EAS vs. SAS	Chr1	1.050	0.950	0.904	0.904	0.950	1.032	0.954	0.954	0.938	0.968	1.023	0.954	0.977	0.954	0.977
	Chr2	1.053	0.947	0.899	0.899	0.947	1.033	0.953	0.953	0.936	0.967	1.024	0.954	0.976	0.954	0.976
	Chr3	1.047	0.953	0.910	0.910	0.953	1.032	0.955	0.955	0.938	0.968	1.023	0.956	0.977	0.956	0.977
	Chr4	1.040	0.960	0.924	0.924	0.960	1.029	0.958	0.958	0.943	0.971	1.023	0.954	0.977	0.954	0.977
	Chr5	1.053	0.947	0.899	0.899	0.947	1.036	0.948	0.948	0.930	0.964	1.028	0.945	0.972	0.945	0.972
	Chr6	1.051	0.949	0.904	0.904	0.949	1.035	0.950	0.950	0.932	0.965	1.029	0.944	0.971	0.944	0.971
	Chr7	1.058	0.942	0.891	0.891	0.942	1.032	0.954	0.954	0.937	0.968	1.021	0.959	0.979	0.959	0.979
	Chr8	1.049	0.951	0.906	0.906	0.951	1.032	0.954	0.954	0.937	0.968	1.015	0.970	0.985	0.970	0.985
	Chr9	1.055	0.945	0.896	0.896	0.945	1.032	0.955	0.955	0.938	0.968	1.020	0.962	0.980	0.962	0.980
	Chr10	1.051	0.949	0.903	0.903	0.949	1.032	0.954	0.954	0.937	0.968	1.022	0.958	0.978	0.958	0.978
	Chr11	1.048	0.952	0.908	0.908	0.952	1.029	0.958	0.958	0.943	0.971	1.019	0.962	0.981	0.962	0.981
	Chr12	1.043	0.957	0.918	0.918	0.957	1.031	0.956	0.956	0.940	0.969	1.026	0.949	0.974	0.949	0.974

	Chr13	1.039	0.961	0.924	0.924	0.961	1.022	0.968	0.968	0.957	0.978	1.018	0.965	0.982	0.965	0.982
	Chr14	1.058	0.942	0.890	0.890	0.942	1.033	0.953	0.953	0.935	0.967	1.024	0.954	0.976	0.954	0.976
	Chr15	1.053	0.947	0.899	0.899	0.947	1.031	0.957	0.957	0.941	0.969	1.023	0.955	0.977	0.955	0.977
	Chr16	1.060	0.940	0.887	0.887	0.940	1.029	0.958	0.958	0.943	0.971	1.009	0.982	0.991	0.982	0.991
	Chr17	1.062	0.938	0.883	0.883	0.938	1.039	0.944	0.944	0.924	0.961	1.028	0.946	0.972	0.946	0.972
	Chr18	1.039	0.961	0.925	0.925	0.961	1.023	0.967	0.967	0.954	0.977	1.020	0.961	0.980	0.961	0.980
	Chr19	1.066	0.934	0.876	0.876	0.934	1.060	0.915	0.915	0.886	0.940	1.050	0.905	0.950	0.905	0.950
	Chr20	1.053	0.947	0.900	0.900	0.947	1.036	0.949	0.949	0.930	0.964	1.019	0.964	0.981	0.964	0.981
	Chr21	1.047	0.953	0.910	0.910	0.953	1.027	0.961	0.961	0.947	0.973	1.022	0.957	0.978	0.957	0.978
	Chr22	1.066	0.934	0.877	0.877	0.934	1.039	0.945	0.945	0.925	0.961	1.024	0.954	0.976	0.954	0.976
	ChrX	1.489	0.511	0.473	0.473	0.511	1.437	0.539	0.539	0.518	0.563	1.430	0.524	0.570	0.524	0.570
	ChrY	1.187	0.813	0.691	0.691	0.813	1.100	0.865	0.865	0.824	0.900	1.075	0.869	0.925	0.869	0.925
Mean across Chromosomes *Per* Population	AFR vs. AMR	1.086	0.914	0.863	0.863	0.914	1.066	0.917	0.917	0.897	0.934	1.060	0.909	0.940	0.909	0.940
	AFR vs. EUR	1.090	0.910	0.858	0.858	0.910	1.070	0.912	0.912	0.891	0.930	1.063	0.903	0.937	0.903	0.937
	AFR vs. EAS	1.079	0.921	0.869	0.869	0.921	1.058	0.925	0.925	0.904	0.942	1.051	0.917	0.949	0.917	0.949
	AFR vs. SAS	1.079	0.921	0.870	0.870	0.921	1.058	0.926	0.926	0.906	0.942	1.051	0.918	0.949	0.918	0.949
	AMR vs. EUR	1.088	0.912	0.862	0.862	0.912	1.064	0.920	0.920	0.900	0.936	1.052	0.921	0.948	0.921	0.948
	AMR vs. EAS	1.085	0.915	0.865	0.865	0.915	1.063	0.921	0.921	0.902	0.937	1.053	0.920	0.947	0.920	0.947
	AMR vs. SAS	1.085	0.915	0.866	0.866	0.915	1.062	0.922	0.922	0.903	0.938	1.051	0.922	0.949	0.922	0.949
	EUR vs. EAS	1.088	0.912	0.860	0.860	0.912	1.065	0.918	0.918	0.898	0.935	1.054	0.918	0.946	0.918	0.946
	EUR vs. SAS	1.086	0.914	0.865	0.865	0.914	1.063	0.921	0.921	0.902	0.937	1.051	0.922	0.949	0.922	0.949
	EAS vs. SAS	1.076	0.924	0.875	0.875	0.924	1.053	0.932	0.932	0.914	0.947	1.042	0.933	0.958	0.933	0.958
Mean across Populations per Chromosome	Chr1	1.053	0.947	0.900	0.900	0.947	1.035	0.950	0.950	0.933	0.965	1.026	0.949	0.974	0.949	0.974
	Chr2	1.055	0.945	0.896	0.896	0.945	1.035	0.950	0.950	0.932	0.965	1.027	0.947	0.973	0.947	0.973
	Chr3	1.048	0.952	0.908	0.908	0.952	1.034	0.952	0.952	0.934	0.966	1.026	0.949	0.974	0.949	0.974
	Chr4	1.042	0.958	0.920	0.920	0.958	1.031	0.955	0.955	0.939	0.969	1.027	0.948	0.973	0.948	0.973
	Chr5	1.053	0.947	0.899	0.899	0.947	1.037	0.948	0.948	0.929	0.963	1.031	0.940	0.969	0.940	0.969
	Chr6	1.053	0.947	0.899	0.899	0.947	1.037	0.948	0.948	0.929	0.963	1.031	0.940	0.969	0.940	0.969
	Chr7	1.059	0.941	0.888	0.888	0.941	1.035	0.950	0.950	0.932	0.965	1.025	0.952	0.975	0.952	0.975
	Chr8	1.051	0.949	0.903	0.903	0.949	1.035	0.950	0.950	0.932	0.965	1.018	0.964	0.982	0.964	0.982
	Chr9	1.056	0.944	0.893	0.893	0.944	1.034	0.952	0.952	0.934	0.966	1.023	0.956	0.977	0.956	0.977
	Chr10	1.052	0.948	0.900	0.900	0.948	1.035	0.951	0.951	0.933	0.965	1.025	0.951	0.975	0.951	0.975
	Chr11	1.050	0.950	0.906	0.906	0.950	1.031	0.956	0.956	0.940	0.969	1.022	0.956	0.978	0.956	0.978
	Chr12	1.046	0.954	0.912	0.912	0.954	1.034	0.952	0.952	0.935	0.966	1.030	0.942	0.970	0.942	0.970
	Chr13	1.041	0.959	0.922	0.922	0.959	1.024	0.966	0.966	0.954	0.976	1.020	0.960	0.980	0.960	0.980
	Chr14	1.060	0.940	0.887	0.887	0.940	1.035	0.950	0.950	0.932	0.965	1.027	0.947	0.973	0.947	0.973
	Chr15	1.054	0.946	0.897	0.897	0.946	1.032	0.954	0.954	0.937	0.968	1.026	0.949	0.974	0.949	0.974
	Chr16	1.062	0.938	0.883	0.883	0.938	1.033	0.953	0.953	0.936	0.967	1.012	0.976	0.988	0.976	0.988
	Chr17	1.063	0.937	0.882	0.882	0.937	1.043	0.939	0.939	0.918	0.957	1.032	0.937	0.968	0.937	0.968
	Chr18	1.041	0.959	0.922	0.922	0.959	1.026	0.963	0.963	0.950	0.974	1.024	0.954	0.976	0.954	0.976
	Chr19	1.065	0.935	0.878	0.878	0.935	1.061	0.914	0.914	0.885	0.939	1.051	0.903	0.949	0.903	0.949
	Chr20	1.054	0.946	0.898	0.898	0.946	1.040	0.943	0.943	0.923	0.960	1.023	0.954	0.977	0.954	0.977

	Chr21	1.047	0.953	0.910	0.910	0.953	1.029	0.959	0.959	0.944	0.971	1.026	0.949	0.974	0.949	0.974
	Chr22	1.065	0.935	0.877	0.877	0.935	1.041	0.942	0.942	0.921	0.959	1.029	0.944	0.971	0.944	0.971
	ChrX	1.497	0.503	0.463	0.463	0.503	1.448	0.527	0.527	0.503	0.552	1.440	0.508	0.560	0.508	0.560
	ChrY	1.351	0.649	0.523	0.523	0.649	1.263	0.686	0.686	0.634	0.737	1.247	0.663	0.753	0.663	0.753

Online Supplementary Figures (Figs S1-S4)

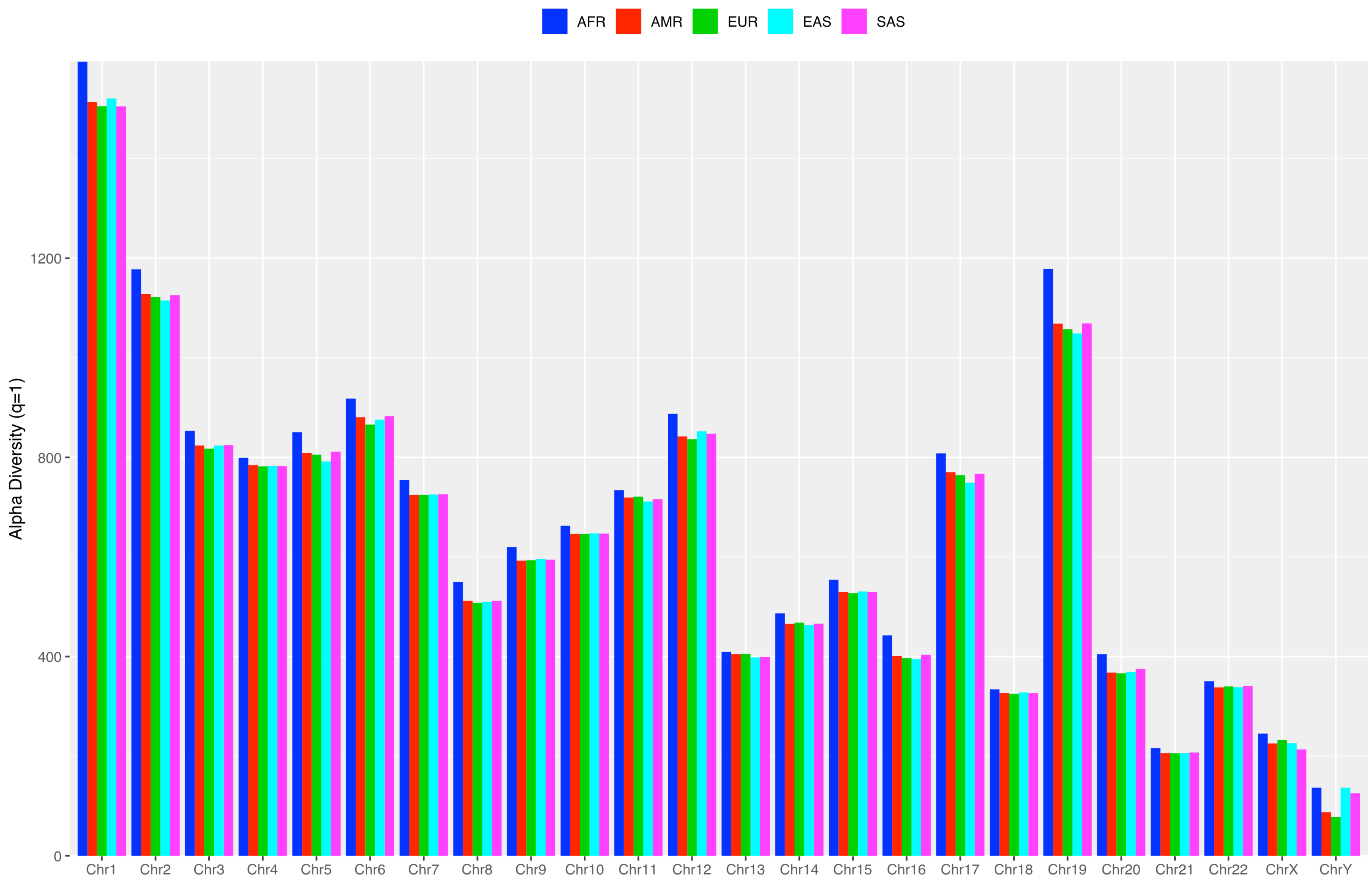

Fig S1. The mean (per individual) SNP alpha-diversity (q=1) at the chromosome level for the 1000-Genomes Project

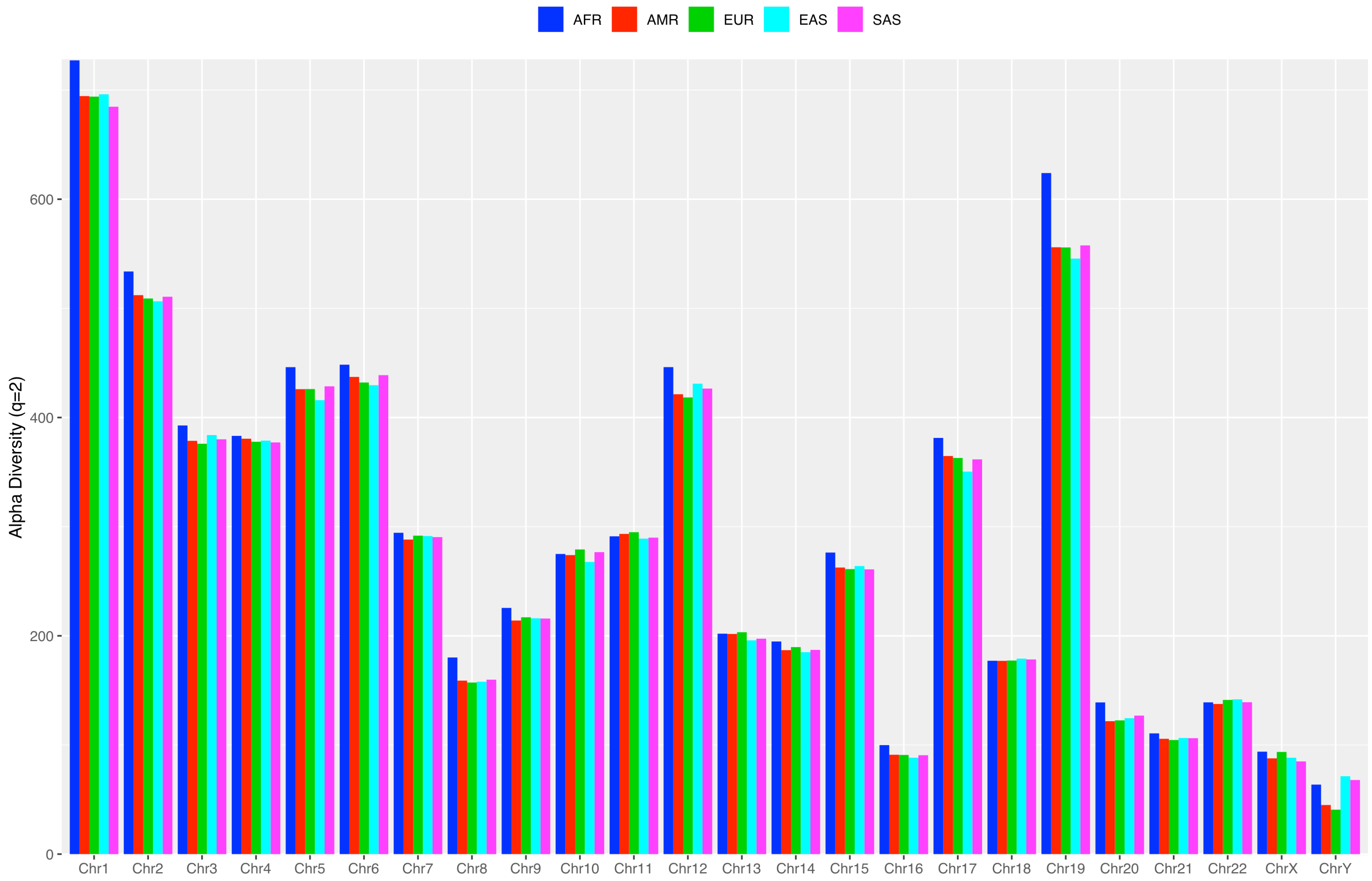

Fig S2. The mean (per individual) SNP alpha-diversity (*q*=2) at the chromosome level for the 1000-Genomes Project

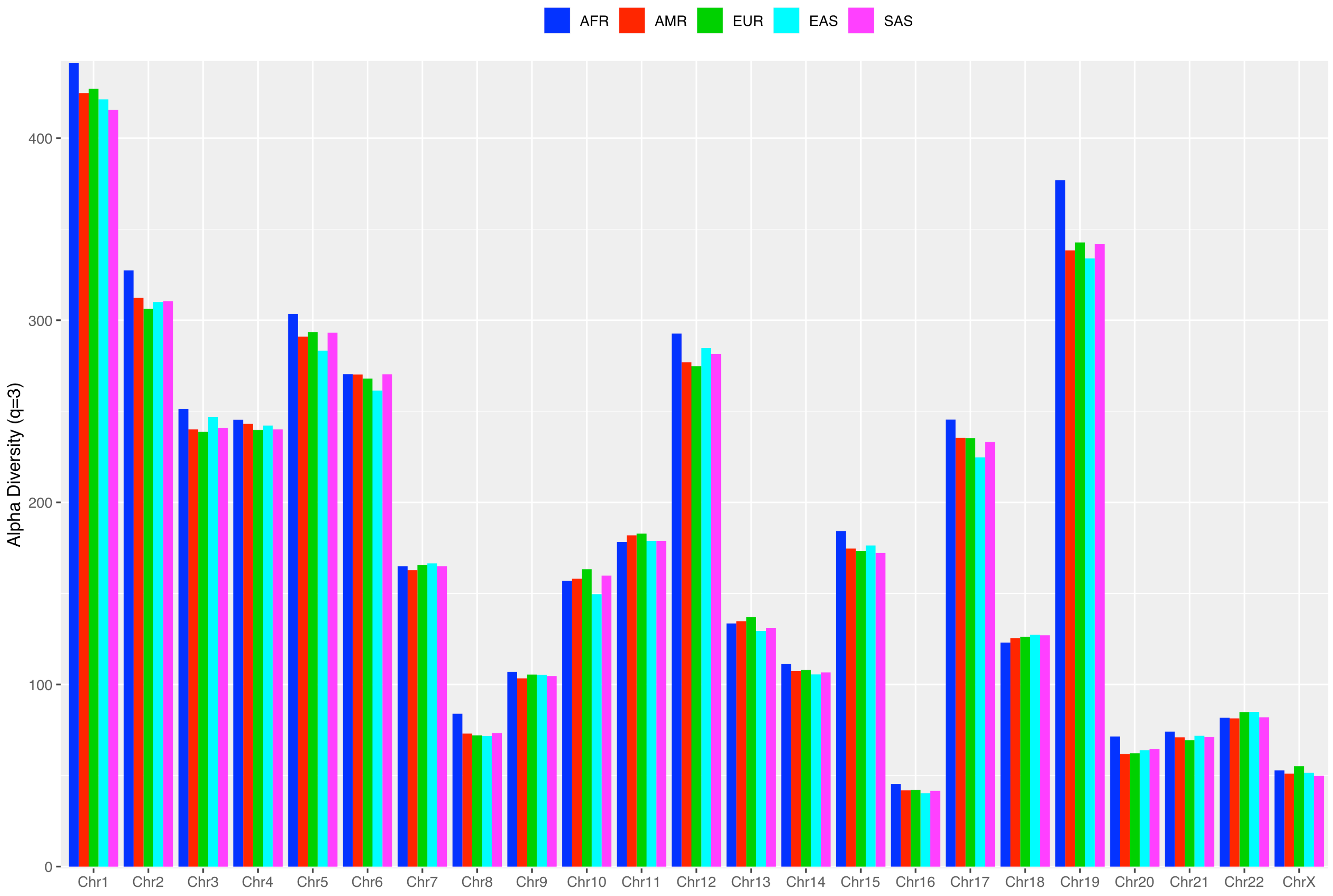

Fig S3. The mean (per individual) SNP alpha-diversity (q=3) at the chromosome level for the 1000-Genomes Project

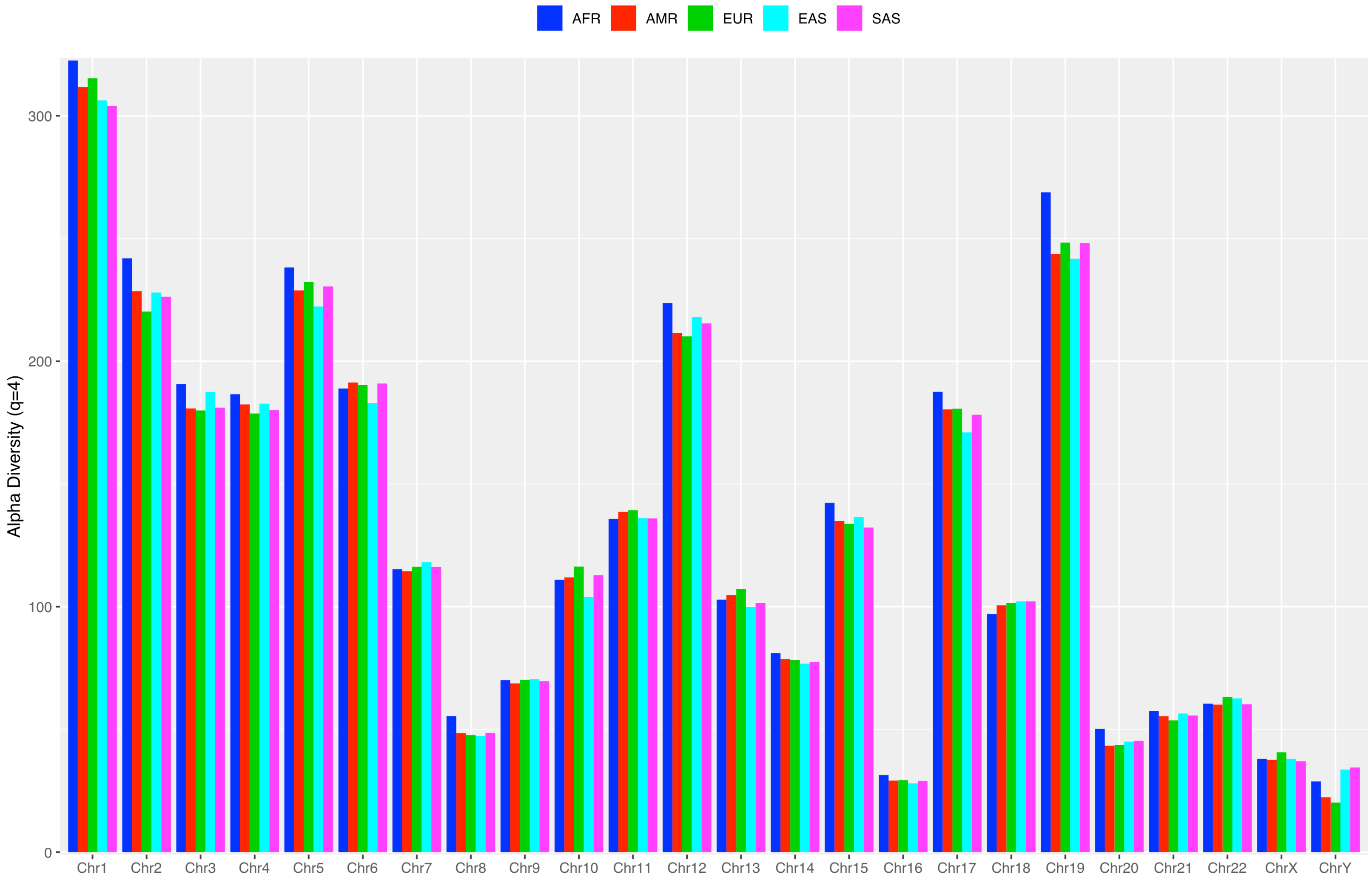

Fig S4. The mean (per individual) SNP alpha-diversity (*q*=4) at the chromosome level for the 1000-Genomes Project

The **R codes** for computing alpha-diversity, beta-diversity (including similarity) profiles

The Help file for R-Codes: `alpha-diversity.r & beta-diversity-similarity.r`

`alpha-diversity.r`

(1) Input file
The input file is an MxN matrix, with elements representing the number of SNPs on each locus from a chromosome or a genome, separated by "\t" (Tab) symbol, where *M* is the number of chromosomes or individuals, and *N* is the number of loci. The first row lists the gene names, and the first column lists sample IDs or names.

(2) Usage
```
$ Rscript alpha-diversity.r filename
```
#filename is the input file containing SNP matrix

(3) Output file
The output file contains the alpha-diversity of each chromosome (or individual). The first column is IDs or names of chromosomes (individuals). The second to the last column are the alpha-diversity values from diversity order (q=0 to q=4).

`beta-diversity-similarity.r`

(1) Input files
Two input files are required to run the code. Each input file is an MxN matrix, with elements representing the number of SNPs on each locus from a chromosome or a genome, separated by "\t" (Tab) symbol, where *M* is the number of chromosomes or individuals, and *N* is the number of loci. The first row lists the gene names, and the first column lists sample IDs or names.

(2) Usage
```
$ Rscript beta-diversity-similarity.r file1 file2
```
file1 and file2 are two files, each of which contains a SNP matrix.

(3) Output file
The output file contains beta-diversity and four similarity indices for each pair-wise chromosome (individual) comparison. The first and second columns are IDs or names of pair-wise individuals. The third column is the diversity order (q). The fourth column is beta-diversity; the remaining columns are the four similarity indices.

```
#SNP alpha-diversity
#usage: Rscript alpha-diversity.r filename    #filesname is the file containing the SNP matrix.

d.chao<-function(A,lev,q)
{
      tot<-sum(A)
      eA<-A/tot
      eA<-eA[eA>0]
      if(is.vector(A)){
            cA<-A
            N<-1
      }else{
            cA<-colSums(A)
            N<-nrow(A)
      }
      ecA<-cA/tot
      ecA<-ecA[ecA>0]
      if(lev=='alpha'){
            if(q!=1){
                  Da<-(1/N)*(sum(eA^q))^(1/(1-q))
                  D.value<-Da
            }else{
                  Da<-exp(-sum(eA*log(eA))-log(N))
                  D.value<-Da
            }
      }
      if(lev=='beta'){
            D.value<-d.chao(A,lev='gamma',q)/d.chao(A,lev='alpha',q)
      }
      if(lev=='gamma'){
            if(q!=1){
                        Dg<-(sum(ecA^q))^(1/(1-q))
                        D.value<-Dg
            }else{
                  Dg<-exp(-sum(ecA*log(ecA)))
                  D.value<-Dg
            }
      }
      D.value
}

# Calculating
args=commandArgs(T)
da=read.table(paste(args[1],sep=""),header=T,sep="\t",stringsAsFactors=F)
ID=as.vector(da[,1])
dat=da[,2:ncol(da)]
dat=as.matrix(dat)
Alpha=matrix(0,nrow(dat),5)
for(n in 1:nrow(dat)){
```

```
		otu=dat[n,]
		otu=otu[otu>0]
		otu=otu[!is.na(otu)]
		for(q in 0:4){
			Alpha[n,q+1]<-d.chao(otu,"alpha",q)
		}
	}
	Alpha=cbind(ID,Alpha)
	colnames(Alpha)=c("ID","q=0","q=1","q=2","q=3","q=4")
	write.table(Alpha,"Alpha-diversity.txt",row.names=F,col.names=T,quote=F,sep="\t")
```

```
# Beta diversity and similarity
# usage: Rscript beta_diversity_similarity.r file1 file2     #file1 and file2 are two files which contain SNP
matrix.

#Function for computing diversity
d.chao<-function(A,lev,q)
{
      tot<-sum(A)
      eA<-A/tot
      eA<-eA[eA>0]
      if(is.vector(A)){
            cA<-A
            N<-1
      }else{
            cA<-colSums(A)
            N<-nrow(A)
      }
      ecA<-cA/tot
      ecA<-ecA[ecA>0]
      if(lev=='alpha'){
            if(q!=1){
                  Da<-(1/N)*(sum(eA^q))^(1/(1-q))
                  D.value<-Da
            }else{
                  Da<-exp(-sum(eA*log(eA))-log(N))
                  D.value<-Da
            }
      }
      if(lev=='beta'){
            D.value<-d.chao(A,lev='gamma',q)/d.chao(A,lev='alpha',q)
      }
      if(lev=='gamma'){
            if(q!=1){
                        Dg<-(sum(ecA^q))^(1/(1-q))
                        D.value<-Dg
            }else{
                  Dg<-exp(-sum(ecA*log(ecA)))
                  D.value<-Dg
            }
      }
      D.value
}

#Functiton for computing similarity: Cq, Uq, Sq, Vq
Cq<-function(A,beta,q,N){
      if(q!=1){
            cq<-(((1/beta)^(q-1))-((1/N)^(q-1)))/(1-(1/N)^(q-1))
      }
      else{
```

```
            tot<-sum(A)
            eA<-A/tot
            cs<-colSums(eA)
            lcs<-log(cs)
            x<-cs*lcs
            x<-x[x!="NaN"]
            hb=-(sum(x))

            pa=A/rowSums(A)
            x<-pa*log(pa)
            y<-rowSums(x,na.rm=T)
            z<-rowSums(A)/tot
            yz=y*z
            x<-yz[yz!="NaN"]
            ha=-(sum(x))

            wa=rowSums(A)/tot
            hc=sum(wa*log(wa))

            cq=(ha-hb-hc)/log(N)
      }
      cq
}

Uq<-function(A,beta,q,N){
      if(q!=1){
            uq<-(((1/beta)^(1-q))-((1/N)^(1-q)))/(1-(1/N)^(1-q))
      }
      else{
            tot<-sum(A)
            eA<-A/tot
            cs<-colSums(eA)
            lcs<-log(cs)
            x<-cs*lcs
            x<-x[x!="NaN"]
            hb=-(sum(x))

            pa=A/rowSums(A)
            x<-pa*log(pa)
            y<-rowSums(x,na.rm=T)
            z<-rowSums(A)/tot
            yz=y*z
            x<-yz[yz!="NaN"]
            ha=-(sum(x))

            wa=rowSums(A)/tot
            hc=sum(wa*log(wa))

            uq=(ha-hb-hc)/log(N)
```

```
        }
        uq
}


Sq<-function(A,beta,q,N){
        sq<-((1/beta)-(1/N))/(1-1/N)
        sq
}

Vq<-function(A,beta,q,N){
        vq<-1-((beta-1)/(N-1))
        vq
}


# Main process
args=commandArgs(T)
dam=read.table(paste(args[1],sep=""),header=T,sep="\t",stringsAsFactors=F)
dan=read.table(paste(args[2],sep=""),header=T,sep="\t",stringsAsFactors=F)
IDm=dam[,1]
IDn=dan[,1]
Res=numeric()
for(x in 1:nrow(dam)){
        for(y in 1:nrow(dan)){
                sda<-rbind(dam[x,2:ncol(dam)],dan[y,2:ncol(dan)])
                for(q in 0:4){
                        beta<-d.chao(sda,"beta",q)
                        cqn<-Cq(sda,beta,q,2)
                        uqn<-Uq(sda,beta,q,2)
                        sqn<-Sq(sda,beta,q,2)
                        vqn<-Vq(sda,beta,q,2)
                        res<-cbind(IDm[x],IDn[y],q,beta,cqn,uqn,sqn,vqn)
                        Res=rbind(Res,res)
                }
        }
}
colnames(Res)=c("ID1","ID2","order","beta.D","cq","uq","sq","vq")
write.table(Res,"Beta-diversity-and-similarity.txt",row.names=F,col.names=T,sep="\t",quote=F)
```
